# Unraveling Autocrine Signaling Pathways through Metabolic Fingerprinting in Serous Ovarian Cancer Cells

**DOI:** 10.3390/biomedicines9121927

**Published:** 2021-12-16

**Authors:** Ji Hee Ha, Muralidharan Jayaraman, Revathy Nadhan, Srishti Kashyap, Priyabrata Mukherjee, Ciro Isidoro, Yong Sang Song, Danny N. Dhanasekaran

**Affiliations:** 1 Stephenson Cancer Center, The University of Oklahoma Health Sciences Center, Oklahoma City, OK 73104, USA; jihee-ha@ouhsc.edu (J.H.H.); muralidharan-jayaraman@ouhsc.edu (M.J.); revathy-nadhan@ouhsc.edu (R.N.); srishti-kashyap@ouhsc.edu (S.K.); priyabrata-mukherjee@ouhsc.edu (P.M.); 2 Department of Cell Biology, The University of Oklahoma Health Sciences Center, Oklahoma City, OK 73104, USA; 3 Department of Pathology, The University of Oklahoma Health Sciences Center, Oklahoma City, OK 73104, USA; 4 Laboratory of Molecular Pathology and NanoBioImaging, Department of Health Sciences, Università del Piemonte Orientale, 28100 Novara, Italy; ciro.isidoro@med.uniupo.it; 5 Department of Obstetrics and Gynecology, Cancer Research Institute, College of Medicine, Seoul National University, Seoul 151-921, Korea; yssong@snu.ac.kr

**Keywords:** ovarian cancer, metabolomics, oncometabolite, glutamate, GABA, 5-HT

## Abstract

Focusing on defining metabolite-based inter-tumoral heterogeneity in ovarian cancer, we investigated the metabolic diversity of a panel of high-grade serous ovarian carcinoma (HGSOC) cell-lines using a metabolomics platform that interrogate 731 compounds. Metabolic fingerprinting followed by 2-dimensional and 3-dimensional principal component analysis established the heterogeneity of the HGSOC cells by clustering them into five distinct metabolic groups compared to the fallopian tube epithelial cell line control. An overall increase in the metabolites associated with aerobic glycolysis and phospholipid metabolism were observed in the majority of the cancer cells. A preponderant increase in the levels of metabolites involved in trans-sulphuration and glutathione synthesis was also observed. More significantly, subsets of HGSOC cells showed an increase in the levels of 5-Hydroxytryptamine, γ-aminobutyrate, or glutamate. Additionally, 5-hydroxytryptamin synthesis inhibitor as well as antagonists of γ-aminobutyrate and glutamate receptors prohibited the proliferation of HGSOC cells, pointing to their potential roles as oncometabolites and ligands for receptor-mediated autocrine signaling in cancer cells. Consistent with this role, 5-Hydroxytryptamine synthesis inhibitor as well as receptor antagonists of γ-aminobutyrate and Glutamate-receptors inhibited the proliferation of HGSOC cells. These antagonists also inhibited the three-dimensional spheroid growth of TYKNU cells, a representative HGSOC cell-line. These results identify 5-HT, GABA, and Glutamate as putative oncometabolites in ovarian cancer metabolic sub-type and point to them as therapeutic targets in a metabolomic fingerprinting-based therapeutic strategy.

## 1. Introduction

Ovarian cancer is the eighth most common cancer in women and ranks fifth in cancer deaths [1,2]. The high mortality rate is largely due to the heterogeneous nature of the disease along with the lack of an efficacious targeted therapy [3,4]. The heterogeneity in ovarian cancer is contributed by histological subtypes of the disease as well as genetic and epigenetic diversity among the tumor cells [5,6]. Recent studies have shown that the intra- and inter tumor differences in metabolic profile also contribute significantly to the heterogeneity of the disease [7]. Metabolic reprogramming is long recognized as one of the hallmarks of cancers and altered cancer metabolome has been associated with aggressive cancer growth and progression [8,9]. Cancer cell metabolome, represented by the net changes in the cancer cell metabolites, is the final iterative outcome from the diverse genomic, transcriptomic and proteomic interactions of the cancer cell [10]. Metabolomics, defined by the “metabolic fingerprinting” of cancer cells, is emerging as one of the central components of precision cancer medicine. It is being increasingly realized that metabolic fingerprinting could provide deeper insights into the metabolic derangements and causative factors, in addition to facilitating the discovery of novel oncometabolites that could serve as new therapeutic targets or prognostic and diagnostic biomarkers in cancer [11,12,13,14,15]. 

With the focus on developing optimal modalities for personalized medicine in ovarian cancer and to identify any oncometabolites that could be targeted for therapy, we carried out metabolic fingerprinting for a panel of high-grade serous ovarian carcinoma (HGSOC) cell lines. The metabolic profiles of fourteen different, commonly used ovarian cancer cell lines, along with control cells represented by normal fallopian tube derived epithelial cells, were obtained using a metabolomic analysis platform that interrogated 731 compounds. Metabolite identification by Mass spectrometry, followed by unsupervised principal component analysis indicated the clustering of the HGSOC cells into five groups. Hierarchical analysis of the results revealed both metabolic convergence and metabolic divergence in the tested cell-lines. Metabolites derived from energy metabolism including aerobic glycolysis and phospholipid metabolisms formed the major basis for convergent commonality among the cancer cells. Convergence towards similarity was also seen in thiol-disulfide redox metabolism as indicated by the increased levels of cysteine metabolites including glutathione. A subset of ovarian cancer cells showed an increase in the levels of glutamate (Glu), γ-aminobutyric acid (GABA), or 5-hydroxytryptamine (5-HT), derived from amino acid metabolism. More importantly, treatment of HGSOC cells with the receptor antagonists or 5-HT synthesis inhibitor attenuated the proliferation of ovarian cancer cells, along with disruption of spheroid formation of these cells. In addition to establishing a metabolome-based sub-classification of HGSOC cells, our results identify Glu, GABA, and 5-HT as oncometabolites that can be effectively targeted by specific receptor- or synthesis-antagonists for therapy in ovarian cancer subtypes.

## 2. Materials and Methods

### 2.1. Cell Lines and Culture

All the cell lines used in this study have been previously described [16,17]. HGSOC cell lines used in this study were obtained from various sources: OVCAR4 cell line was obtained from Dr. Thomas Hamilton (Fox Chase Cancer Center, Philadelphia, PA, USA) and OVCAR8 cells were from National Cancer Institute (NCI). Kuramochi, TYKNU, OVKATE and OVSAHO cell lines were from the JCRB Cell Bank, Tokyo, Japan. SNU119, SNU251, ES-2, OVCAR3, CAOV3 and OV90 cells were from Seoul National University, Seoul, Korea, and COV362, OAW28 and COV318 cells were purchased from Sigma-Aldrich (St. Louis, MO, USA). All cell lines were authenticated by short tandem repeat analysis [16]. OVCAR3, OVCAR8, OVKATE, SNU119, SNU251, OVCAR4, OVSAHO, and Kuramochi cells were maintained in Roswell Park Memorial Institute (RPMI) 1640 medium (Cellgro, Manassas, VA, USA); COV362, COV318, OAW28 and CAOV3 cells were maintained in Dulbecco’s modified Eagle’s (DMEM) Medium (Cellgro, Manassas, VA, USA); OV90 cells were maintained in MCDB105:M199 (1:1) Medium (Thermo Fisher Scientific, Waltham, MA, USA); TYKNU cells were maintained in Minimum Essential Medium (MEM) (Cellgro, Manassas, VA, USA); and ES-2 cells were maintained in McCoy’s 5A medium (Sigma-Aldrich, St. Louis, MO, USA). All cells were maintained at 37 °C in a 5% CO_2_ incubator. All media were supplemented with 10% FBS (Gemini Bio-Products, West Sacramento, CA, USA), 50 U/mL penicillin, 50 μg/mL streptomycin (Cellgro, Manassas, VA, USA). Glutamate-receptor antagonist, MK801 (Cat # S2876) and GABA_A_-receptor antagonist, Bicuculline (Cat # S7071) were obtained from Selleckchem (Houston, TX, USA). GABA_B_-receptor antagonist, CGP55845 (Cat # 1248) was procured from Tocris Bioscience (Minneapolis, MN, USA) whereas tryptophan hydroxylase inhibitor, LX-1031 (Cat # ab269814) was purchased from Abcam (Waltham, MA, USA). Glutamate-receptor antagonist, MK801, GABA_A_-receptor antagonist, Bicuculline, GABA_B_-receptor antagonist, CGP55845, and tryptophan hydroxylase inhibitor, LX-1031 were dissolved and stored in DMSO as 10 mM, 50 mM, 50 mM, and 25 mM stock, respectively, until use.

### 2.2. Metabolomic Analysis

Cells were synchronized by serum starvation for 18 h following our previously published methods [18] and stimulated to proliferate with the addition of serum. Actively growing cells at 14 h following serum stimulation were pelleted. Cells were grown in triplicates per cell line. Two of the triplicate cell pellets (per each cell line) representing 14 HGSOC cell lines along with FTE188 control cells were subjected to metabolomic analysis. Frozen pellets were transferred to Metabolon, Inc. (Morrisville, NC, USA) for metabolomic analysis. Non-targeted metabolomic analysis was carried out by Metabolon, Inc. (Morrisville, NC, USA) as described previously [19]. Sample preparation, extraction, liquid chromatography, and mass spectrometry were carried out following the methods established by Metabolon, Inc. as presented under “Supplemental Methods”. Briefly, global metabolic profiles were determined using a Waters ACQUITY ultra-performance liquid chromatography (UPLC) and a Thermo Scientific Q-Exactive high resolution/accurate mass spectrometer interfaced with a heated electrospray ionization (HESI-II) source and Orbitrap mass analyzer operated at 35,000 mass resolution. Appropriate quality control samples (Table S1) and quality control stndarads (Table S2) were used to ascertain instrument variability and total process variability (Table S3). Compounds were detected and identified by comparison to library entries of purified standards or recurrent unknown entities using Metabolon’s proprietary hardware and software. The data were normalized with respect to protein concentration of the respective cell pellets.

### 2.3. Data Processing

Raw data were extracted, peak-identified and QC processed using Peaks were quantified using area-under-the-curve method utilizing ion counts for relative quantification. Metabolites that did not register in >80% of the samples were removed. The remainder missing values were imputed with the value equivalent to 50% of the minimal value in the raw data and considered as missing data below the detection limit. The values of the metabolites in the raw data that registered below 25% in the interquartile scale were considered near constant and removed. The present dataset comprises a total of 731 compounds of known identity (Supplementary Table S4). Total number of metabolites examined and their unique chemical class identifier are presented in Supplementary Table S5. Detailed methodology including statistical and bioinformatic analyses of the data are included in the supplemental material (Supplemental Methods).

### 2.4. Statistical Analysis

For cell line-based in vitro analyses, two-way ANOVA was carried out to determine the interaction effects of inhibitor and incubation period on the cell proliferation. Further, a Dunnett’s multiple comparison post-hoc test between the control and each concentration of the inhibitor at each time point was performed. Results were presented as bara graphs of percent inhibition over control with the significance represented using the p-values from the post-hoc test. For metabolomic analysis, two types of statistical analysis were carried out: (1) significance tests and (2) classification analysis. Standard statistical analyses were performed in ArrayStudio version 7.2 (Qiagen OmicSoft, Cary, NC, USA) on log transformed data. The data from the experimental groups were compared using Multivariate ANOVA. For the analyses not standard in ArrayStudio, the programs R (http://cran.r-project.org/; accessed on 13 January 2020) or JMP software (JMP Inc., Cary, NC, USA) were used. Post-hoc *p*-values and false discovery rates were calculated by determining the *p*- and *q*-values using Storey’s method [20]. Only the data with the *p*-value of <0.05 were considered significant. Quality control samples, quality control standards, FDR values of <0.05 were considered significant. Complete dataset with *p*- and *q*-values are deposited at the NIH Common Fund’s National Metabolomics Data Repository (https://www.metabolomicsworkbench.org/data/DRCCMetadata.php?Mode=Project&ProjectID=PR001259; accessed on 13 January 2020). 

### 2.5. Principal Component Analysis and Hierarchical Clustering

Principal component analysis (PCA) for high dimension and multivariate data was carried out with two or three principal components. Each principal component is a linear combination of every metabolite and the principal components are uncorrelated. The first component was computed by determining the coefficients of the metabolites that maximizes the variance of the linear combination. The second principal component is computed by determining the coefficient that maximized the variance in which the second component is orthogonal to the first. The third component is defined by the coefficient of variance, but orthogonal to the first two components. Unsupervised hierarchical clustering was carried out by hierarchical clustering using Euclidean distance methods. Each cell line sample is considered as a vector with all of the metabolic values.

### 2.6. Cell Proliferation and InhibitorAnalysis

Kuramochi, SNU119, TYKNU, COV362, or OV90 cells were seeded into 96-well plates (1 × 10^4^ cells/well) in appropriate growth media and incubated in Sartorious IncuCyte^®^ S3 Live-Cell Analysis System (37 °C; 5% CO_2_). At 24 h, the cells were treated with varying concentrations of each of the inhibitors (10 μM and 25 μM) or the vehicle, DMSO (0.05% final concentration/well). The doses of the inhibitors were ascertained based on initial dose optimizations. Their effects on cell proliferation were monitored by imaging the cells at 12 h intervals. At each time point, images were taken from eight random wells (4 imgaes/well/group) in the phase bright-field channel at 10× magnification. Cell proliferation was monitored by analyzing the occupied area (Confluence %) of cell images over time, using Cell-by-Cell Analysis Software Module of the IncuCyte^®^ GUI 2020A software using Adherent Cell-by-Cell Analysis methodology.

### 2.7. 3D-Spheroid Analysis

The 3D spheroid growth analysis was carried out following the previously published methods [21]. Cells were plated at the density of 7 × 10^3^ cells/200 μl per well in 96-well Corning Round bottom ULA plates (Corning, Corning, NY, USA) and incubated in IncuCyte S3 Live-Cell Analysis system. At 24 h, the cells were treated with the vehicle or the antagonists (10 µM) and spheroid growth was monitored by imaging the cells at 12 h-intervals for 6 days. Images were acquired at 4x magnification. Spheroid formation was quantified in terms of total spheroid area (µm^2^) using Incucyte S3 Spheroid Software Module.

## 3. Results

### 3.1. Metabolomic Heterogeneity and Clustering of HGSOC Cell Lines

**Figure 1:**
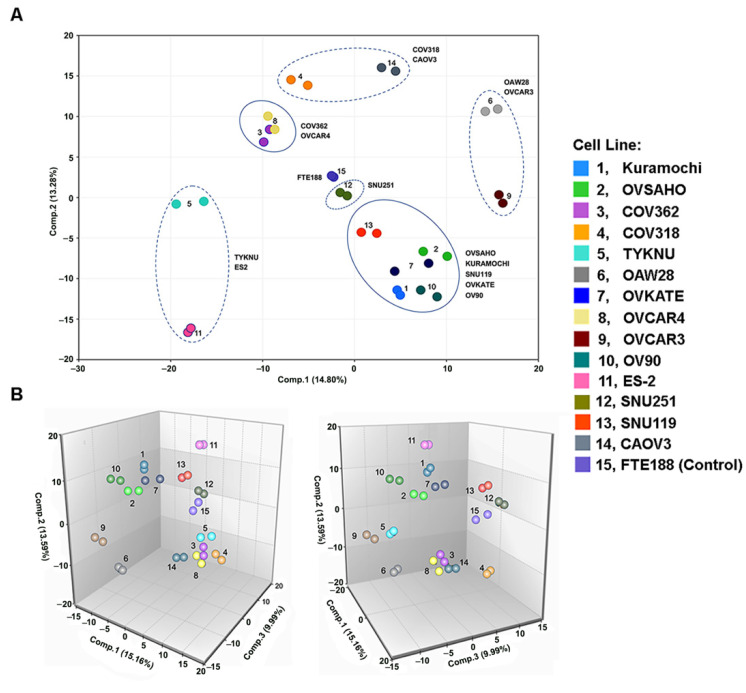
PCA analysis of metabolites in HGSOC cell lines. Two-dimensional (**A**) as well as three-dimensional PCA (**B**) score plot of the metabolomic profiles of the HGSOC cell lines along with the control cell line FTE188 is presented. (**A**). X and Y axis of the plot denotes the percentile variations defined by principal component-1 and principal component-2, respectively. (**B**). Percentile variations defined by principal component-1, principal component-2, and principal component-3 are indicated in the X, Y, and Z axis of the plot. Numbered and color-coded dots represent the individual cell lines as indicated in the side panel. Clustering of the HGSOC cells based on the metabolomic profiles is circled. Solid circles indicate closely related clusters whereas dotted circles indicate distantly related clusters. Cell lines that constitute the respective clusters are indicated.

A total of 731 metabolites of known identity was analyzed for their presence in fourteen HGSOC cell lines and the control Fallopian Tube-derived epithelial cell line, FTE188. Lysates from these cells were subjected to Ultrahigh Performance Liquid Chromatography-Tandem Mass Spectroscopy. The high-dimensional dataset for each of the cell lines was obtained following metabolite identification, metabolite quantification, and data normalization. An unsupervised principal component analysis (PCA) was carried out using all the samples to determine whether the ovarian cancer cell lines can be segregated from the FTE188 control and from each other, based on differences in their overall metabolite signature. Two-dimensional (2D-PCA) as well as three-dimensional principal component analysis (3D-PCA) was carried out. Both the PCA plots indicated that the global metabolite profiles of the cancer cells differed widely from the control cells (Figure 1A,B). 

It is pertinent to note here that the reproducibility among the biologically duplicate samples was indicated by the tight clustering between the duplicates. More significantly, the PCA results indicated the substantial differences in the metabolite profiles of the different cell lines. The simplest analysis using 2D-PCA clearly indicated that the global metabolite profile of SNU251 cancer cells appeared to be very similar to the FTE188 control. Whereas, ES-2, OVCAR3, and OAW28 cell lines displayed the greatest differences from the control cell line (Figure 1A). These results also indicated the potential similarities in the metabolomic profiles of COV318, COV362, and OVCAR4 cell lines, which formed a metabolically-related cluster of HGSOC cells (Figure 1A). This overall metabolomic clustering of HGSOC cell lines, was further corroborated by the plots derived from 3D-PCA analysis (Figure 1B). 

**Figure 2:**
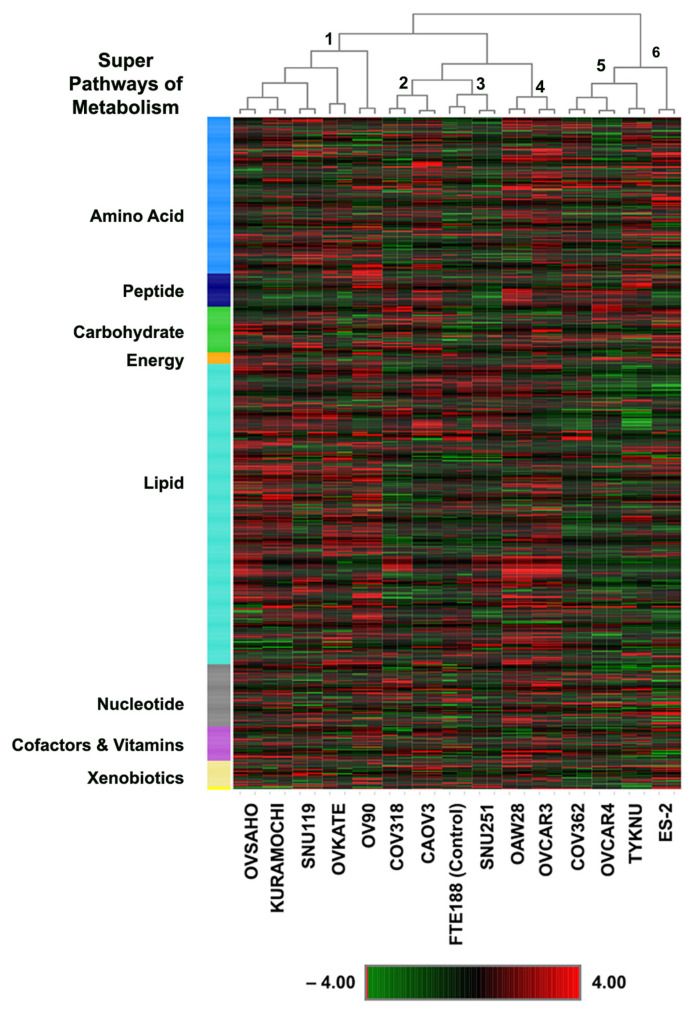
Heatmap of differential metabolites in HGSOC cell lines. Similarities and differences between metabolite profiles were analyzed by hierarchical clustering using Euclidean distances derived from the group means. Reproducibility of the duplicate samples is indicated by the tight clustering of the duplicate samples. The color scale from green to red corresponds to low to high concentrations in log scale. The clustering of the HGSOC into six clusters is denoted by numbers in the clades of the dendrogram.

To gain more granular details on the similarities and differences in the metabolomic profiles of the HGSOC cells, the dataset was analyzed by unsupervised hierarchical clustering (Figure 2). 

**Figure 3:**
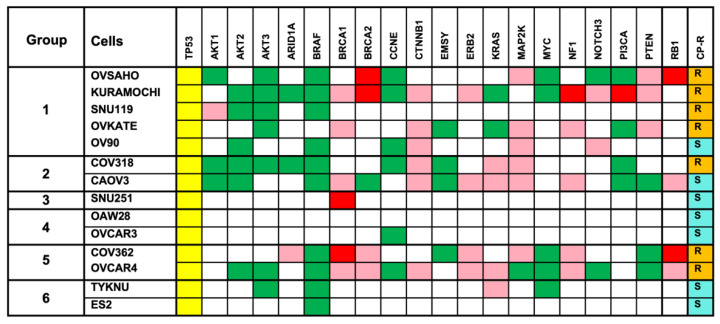
Metabolome-based Clustering of HGSOC Cells. Based on PCA and hierarchical analysis of the metabolomic profiles, fourteen different HGSOC cell lines are clustered into six metabolomic clusters. Known mutational profiles are indicated by differently colored squares: Yellow, Deletion or loss of function mutation; Pink, Heterozygous loss; Red, amplification or gain of function mutation; and Green, loss of function mutation. The column labeled CP-R refers to cisplatin-resistance with R and S indicating the resistance and sensitive phenotypes of the cells.

Results from hierarchical clustering validated the PCA results to a large extent. As observed in the results from PCA analyses (Figure 1A,B), replicate sample pairs were tightly clustered. The dendrogram from the dataset clustered the HGSOC cells into two major branches or clades. OVSAHO, Kuramochi, SNU119, OVKATE, OV90, COV318, CAOV3, SNU25, OAW28, and OVCAR3 clustered into one clade, whereas COV362, OVCAR4, TYKNU, and ES2cells clustered into the second clade. The first clade branched out into four minor clades whereas the second clade bifurcated into two minor clades (Figure 2). Similar to the results from PCA analysis, SNU251 cell clustered next to the FTE188 control. Thus, based on the dendrogram structure, the HGSOC cells can be grouped into six metabolic sub-types as depicted in Figure 3. 

### 3.2. Glycolysis and TCA Metabolism Profile in Ovarian Cancer Cells

**Figure 4:**
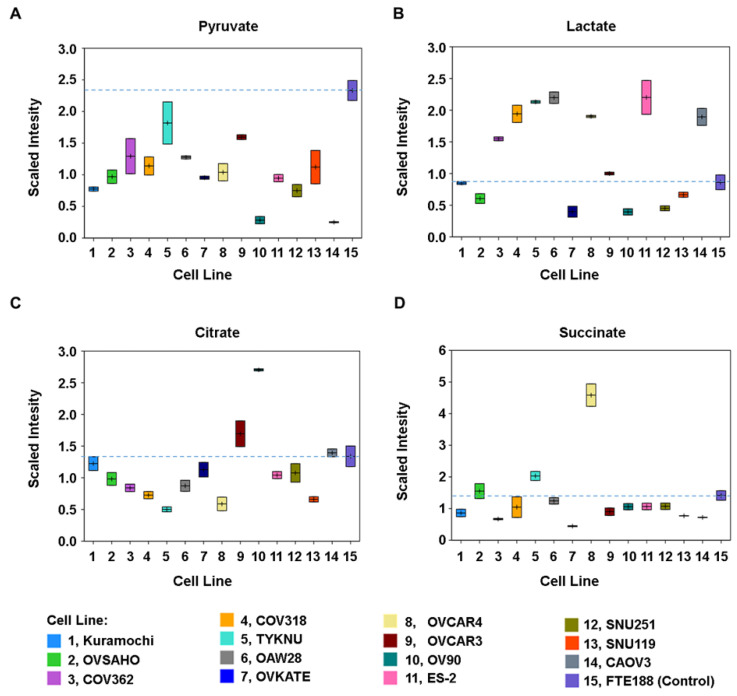
Profiles of central energy metabolism metabolites in HGSOC cells. Changes in key metabolites that serve as branching points in central energy metabolism are profiled. Relative contents of the respective metabolites are expressed as scaled intensity in the y-axis and the cell types are presented in the x-axis. Scaled intensity is an arbitrary unit relative to the overall median of 1 for the test metabolite. Data points are presented as boxes in the box plot in which the center line in boxes in denotes the median value. The upper and lower borders define the two measurements of scaled intensity as described under Supplemental Methods. (**A**). Changes in the levels of pyruvate that could enter into aerobic glycolysis as well as TCA cycle pathways were quantified. (**B**). Increased levels of lactate typically indicating increased glycolysis, and/or reduced conversion of pyruvate to acetyl-CoA, for entry into the TCA cycle were determined. (**C**). Changes in the TCA-cycle metabolites, represented by citrate (**C**) and succinate (**D**), were also monitored.

Cancer cells derive energy for their survival and growth through the stimulation of both oxidative phosphorylation and aerobic glycolysis. This involves the uptake of glucose and subsequent breakdown of glucose into two molecules of pyruvate, with the release of 2 molecules of ATP. Consistent with this metabolic framework, glucose levels were found to be higher in all cancer cells, relative to control, to different degrees (Figure S1). In addition, increased levels of Glucose 6-phosphate, hexose diphosphate, and phosphoenolpyruvate were seen in most of the cancer cells (Figure S1). Pyruvate, thus formed, is rapidly utilized for the synthesis of lactate, acetyl CoA, or Alanine through different mechanisms. Our results, as presented in Figure 4, validates such metabolic rewiring that favors aerobic glycolysis. Pyruvate levels were observed to be lower in all the cancer cell lines relative to control, indicating its rapid turnover (Figure 4A). More interestingly, lactate levels were elevated in COV362, OVSAHO, TYKNU, OAW28, OVCAR4, ES2, and CAOV3 cells (Figure 4B). Increased lactate levels typically represent increased glycolysis or reduced conversion of pyruvate to acetyl-CoA for entry into the TCA cycle. Citrate, which is formed from the condensation of oxaloacetate and acetyl-CoA was lower in a majority of the tumor lines, excepting OVCAR3 and OV90 (Figure 4C). Succinate, which is downstream of citrate in the TCA cycle showed such a decrease in the majority of the cancer cells, with the exception of TYKNU and OVCAR4 (Figure 4D). Thus, the glycolysis and TCA cycle metabolite data suggest that the oxidative phosphorylation in suppressed in majority of the ovarian cancer cells in favor of aerobic glycolysis.

### 3.3. Increased Synthesis and Turnover of Phospholipids in HGSOC Cells

**Figure 5:**
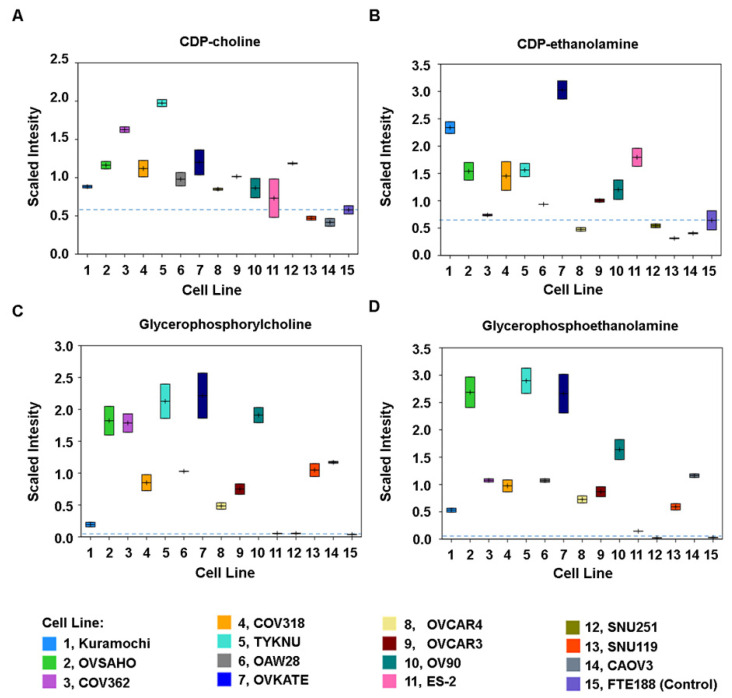
Higher Phospholipid Synthesis and Turnover in HGSOC Cells. Phospholipid synthesis and turnover are indicated by levels of the key branch-point metabolites. Relative contents of the respective metabolites are expressed as scaled intensity in the y-axis against the cell types presented in the x-axis. Data points are presented as boxes in the box plot in which the center line in boxes in denotes the median value. The upper and lower borders define the two measurements of scaled intensity. Changes in overall phospholipid synthesis was monitored by CDP-Choline (**A**) and CDP-ethanolamine levels (**B**). Changes in phospholipid degradation was monitored by glycerophosphocholine (**C**) and glycerophosphoethanolamine (**D**) levels.

Phospholipids formed another group of metabolites that showed deviation from the non-malignant control cells (Figure 4). Phospholipids are synthesized by conjugating choline or ethanolamine head groups to diacylglycerols with the generation of cytidine diphosphocholine (CDP-choline) or cytidine diphosphoethanolamine (CDP-ethanolamine). While the individual phospholipid levels were not consistently changed in each of the cancer cells, the precursors to phospholipid synthesis such as CDP-Choline and CDP-ethanolamine, were sharply elevated in many of the cancer lines (Figure 5A,B). In addition, higher levels of glycerophosphoethanolamine and glycerophosphocholine were determined in a majority of cancer cells (KURAMOCHI, OVSAHO, COV362, COV318, OAW28, TYKNU, OVKATE, OVCAR4, OVCAR3, OV90, SNU119, CAOV3) excepting ES2 and SNU251 cells, indicating an increased turnover of phospholipids in these cells (Figure 5C,D).

### 3.4. Increased Amino Acid Metabolism in HGSOC Cells

**Figure 6:**
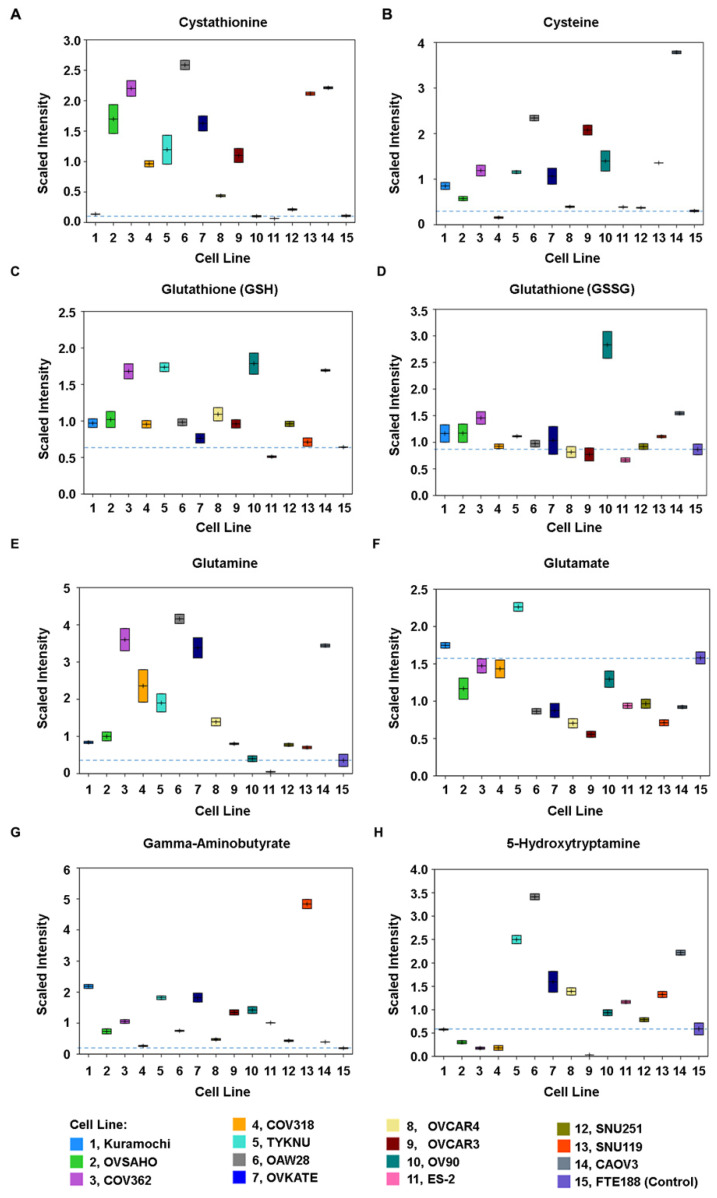
Changes in Amino Acid Metabolism. Changes in the metabolomic profile involving Cysteine, Glutamine, and Tryptophan were monitored. Relative contents of the respective metabolites, expressed as scaled intensity, were plotted in the *y*-axis against the cell types plotted in the *x*-axis. Data points are presented as boxes in the box plot in which the center line in boxes in denotes the median value. The upper and lower borders define the two measurements of scaled intensity. Levels of cystathionine (**A**) that acts as precursor for cysteine and cysteine (**B**) were monitored. Changes in the profiles of reduced glutathione (**C**) and its oxidized GSSG (**D**) counterpart were quantified. Differences in the levels of Glutamine (**E**). and Glutamate (**F**) were also profiled. Levels of GABA (**G**) and 5-HT (**H**) in different HGSOC cells were also determined.

Results from the metabolic fingerprinting of ovarian cancer cells also indicated an upregulation in the metabolic pathways involving the metabolism of several amino acids (Figure 5). An increase in the levels of intermediary compounds in the methionine-cysteine-glutathione synthetic pathway was observed. Levels of cystathionine, a precursor of cysteine (Cys), was found to be higher in most of the cancer cells excepting Kuramochi, OV90, ES-2, and SNU251 cells (Figure 6A). A similar increase in the levels of Cys was also observed in these cells (Figure 6B). Correlating with the precursor role of Cys in glutathione synthesis, reduced-glutathione (GSH) also showed increased levels in many of the cancer cells (Figure 6C). Relative increase in the levels of glutathione-GSH over oxidized-glutathione (GSSG) was detected in COV362, TYKNU, and CAOV3 cells (Figure 5C,D). Increase in the levels of both glutathione-GSH and glutathione-GSSG was observed in OV90 cells (Figure 6C,D).

In addition to Cys metabolism, cancer cells showed perturbations in glutamine (Gln) and glutamate/glutamic acid (Glu) metabolism. All of the HGSOC cells—barring ES2 cells—showed an increase in Gln levels (Figure 6E). Gln has been known to play a key role in cancer cell growth and energy metabolism. Gln utilization by the cells involve the conversion of Gln into Glu by glutaminases. Although increased Glu levels were not seen in many of the HGSOC cells, excepting Kuramochi and TYKNU cells (Figure 6F), it should be noted that Glu is rapidly metabolized in the cell and utilized as a substrate in multiple pathways involved in energy metabolism and macromolecular synthesis [22]. One such major pathway involves the conversion of Glu into the neurotransmitter γ-aminobutyrate or γ-aminobutyric acid (GABA) by glutamic acid decarboxylase. Interestingly, HGSOC cells showed an increase in the levels of GABA (Figure 6G). 

Majority of the ovarian cancer cells also showed an increase in the levels of tryptophan, suggesting an enhanced uptake of this amino acid by the cancer cells (Figure S1). Tryptophan can be readily metabolized into 5-hydroxytryptamine, kynurenine, or indole pyruvate by three different enzymatic processes. While a few of the cells exhibited increased levels of kynurenine (Figure S1), 9 out of 14 cell lines showed an increase in 5-HT levels (Figure 6H). 

GABA, or 5-HT (Figure 6E–H). GABA, Glu, and 5-HT have been well characterized as neurotransmitters as well as neurotrophic factors that activate intracellular signaling pathway via the stimulation of specific sets of cell surface receptors. Evidence is emerging that they can elicit diverse non-neuronal response by stimulating their receptors in non-neuronal tissues and recent studies have shown that the extra-neuronal synthesis and release of neurotransmitters cells into the TME by the cancer cells promote tumorigenesis and tumor progression in many cancers [23,24]. Based on previously reported pro-tumorigenic signaling roles of 5-HT [23,25,26,27], GABA [23,28,29], and Glutamate [22,23,30] in many other cancer cells, we sought to test their potential role as oncometabolites in HGSOC cells.

### 3.5. Targeting the Potential GABA-Mediated Autocrine Signaling in HGSOC Cells

**Figure 7:**
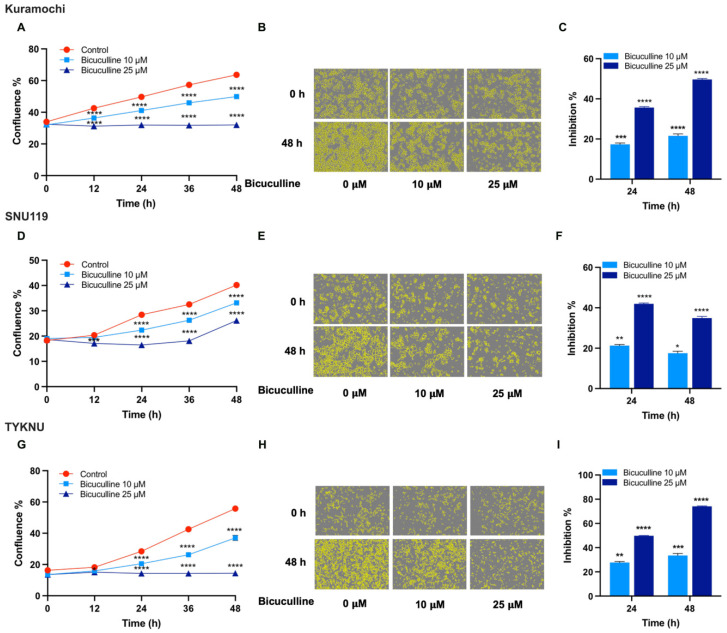
Inhibition of Ovarian Cancer Cell Proliferation by Bicuculline, GABA_A_-receptor Antagonist. Kuramochi, SNU119, and TYKNU cells were treated with vehicle control or Bicuculline at the concentrations of 10 µM and 25 µM and monitored for 48 h in IncuCyte S3 Live-Cell Analysis system for cell proliferation at 12 h-intervals for 48 h, as described under Methods. (**A**,**D**,**G**). Cell proliferation is expressed as confluence % of the cells. (**B**,**E**,**H**). Representative phase contrast bright field micrographs of cells with cell masks (yellow) at 48 h (10× magnification) are presented. (**C**,**F**,**I**). Percent inhibitions over the untreated control levels were quantified and presented. The experiments were repeated three times and the results are from a representative experiment. Error bars represent mean ± SEM (n = 8). Statistical significance between the inhibitor treated groups and the untreated control at each time pint was determined by two-way ANOVA and Dunnett’s multiple comparison post-hoc test (*, *p* < 0.05; **, *p* < 0.005; ***, *p* < 0.0005; ****, *p* < 0.0001).

It is of interest to note here that GABA levels have been shown to be elevated in the urine of ovarian cancer patients suggesting a pathological role for increased GABA levels in ovarian cancers [31]. GABA has been shown to promote the proliferation of diverse cancer cells including those of gastric cancer and pancreatic adenocarcinoma through the activation of ionotropic GABA_A_ receptors [28,32]. Therefore, a panel of ovarian cancer cells—Kuramochi, TYKNU, and SNU119—that showed increased levels of GABA were treated with two different concentrations of bicuculline, a GABA_A_-receptor antagonist. Proliferation of the cells were monitored using Incucyte live cell kinetic proliferation assay. Treatment of cells with bicuculline drastically inhibited the proliferation of the HGSOC cells in a dose-dependent manner (Figure 7). 

**Figure 8:**
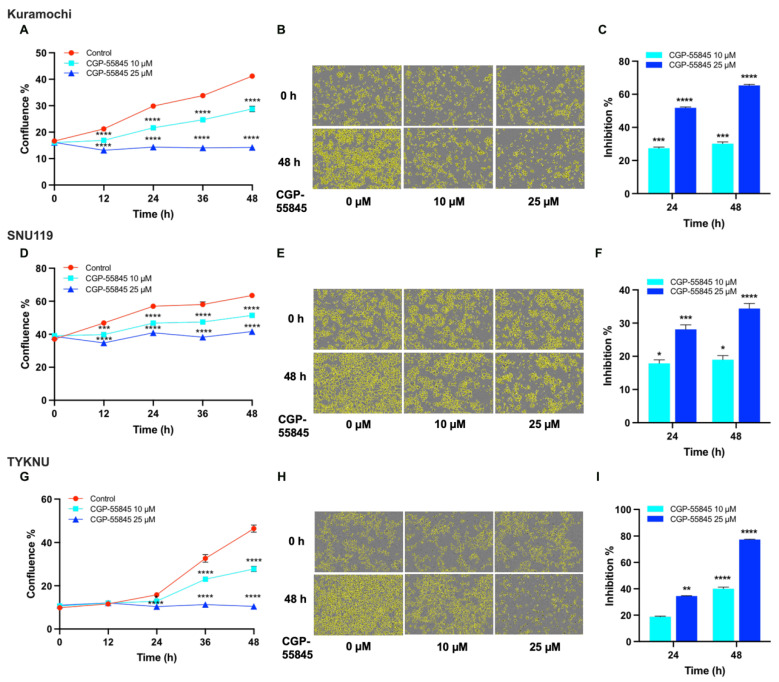
Inhibition of Ovarian Cancer Cell Proliferation by CGP-55845, GABA_B_-receptor Antagonist**.** Kuramochi, SNU119, and TYKNU, ovarian cancer cells were treated with vehicle control or CGP-55845 at concentrations of 10 µM and 25 µM. Cell proliferation was monitored for 48 h in IncuCyte S3 Live-Cell Analysis System. (**A**,**D**,**G**). Cell proliferation is expressed as confluence % of the cells; (**B**,**E**,**H**). Phase contrast bright field micrographs of cells with cell masks (yellow), at 48 h, 10× magnification are presented; and (**C**,**F**,**I**). Percent inhibitions over the vehicle control were quantified and presented. The experiments were repeated three times, and the results are from a representative experiment. Error bars represent mean ± SEM. Statistical significance between the inhibitor treated groups and the vehicle treated control at each time point was determined by two-way ANOVA and Dunnett’s multiple comparison post-hoc test (*, *p* < 0.05; **, *p* < 0.005; ***, *p* < 0.0005; ****, *p* < 0.0001).

It has also been shown that GABA can stimulate the proliferation of prostate cancer cells via the activation of metabotropic GABA_B_ receptors [33]. Therefore, the panel of HGSOC cells mentioned above were also tested for their sensitivity to GABA_B_-receptor antagonist, CGP-55845. Cells were treated with two different concentrations of CGP-55845 and the proliferation of the cells was monitored. As shown in Figure 8, treatment of cells with CGP-55845 potently inhibited the proliferation of the HGSOC cells in a dose-dependent manner similar to GABA_A_-receptor antagonist.

### 3.6. Targeting the Potential Glu-Mediated Autocrine Signaling in HGSOC Cells

**Figure 9:**
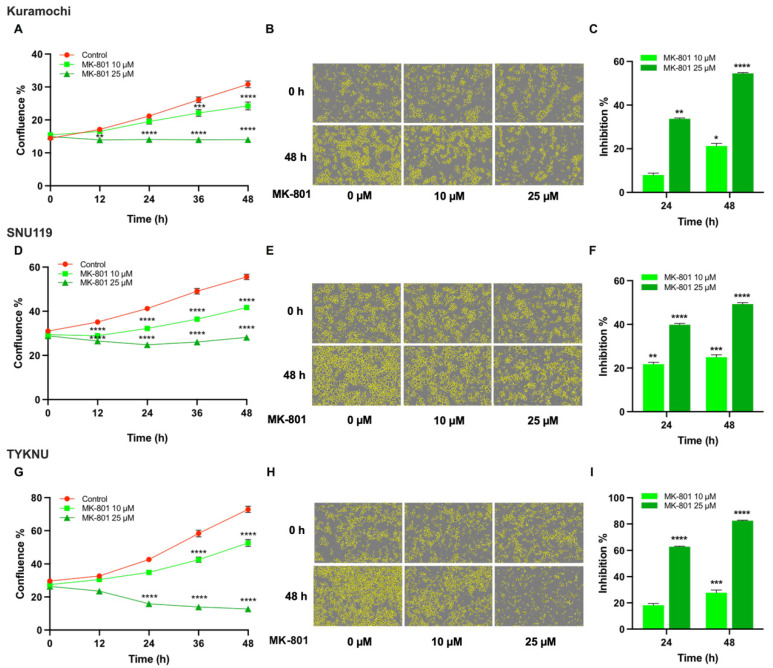
Inhibition of Ovarian Cancer Cell Proliferation by MK-801, Glu/NMDA-receptor Antagonist. Kuramochi, SNU119, and TYKNU, cells were seeded treated with vehicle control or MK-801, a Glu/NMDA receptor antagonist, at concentrations of 10 µM and 25 µM and cell proliferation was monitored for 48 h in IncuCyte S3 Live-Cell Analysis System. (**A**,**D**,**G**). Cell proliferation was expressed in terms of total confluence % of the cells; (**B**,**E**,**H**). 48 h-Phase contrast bright field micrographs of cells with cell masks (yellow), at 10× magnification are presented; (**C**,**F**,**I**). Percent inhibitions over the untreated control were quantified. Presented results are from a typical experiment (n = 3). Error bars represent mean ± SEM. Statistical significance between the inhibitor treated groups and the untreated control at each time point was determined by two-way ANOVA and Dunnett’s multiple comparison post-hoc test (*, *p* < 0.05; **, *p* < 0.005; ***, *p* < 0.0005; ****, *p* < 0.0001).

Potential role of glutamate as an oncogenic growth factor has been demonstrated using breast, lung, or pancreatic cancer cells [34]. However, its role in ovarian cancer is far from clear. Among the different metabotropic- (mGluR) and ionotropic- (iGluR) glutamate receptors (GluR), the iGluRs belonging to N-methyl-D-aspartate receptor (NMDAR) family are gaining importance as targets in cancer, primarily due to their ability to stimulate mTOR- and ERK-signaling pathways that could be correlated with cell survival and proliferation [35,36,37]. Previous studies have demonstrated the increased expression of NMDAR subunits in ovarian cancer tissues and the ability of NMDAR-antagonists to inhibit the proliferation of non-serous ovarian cancer cell lines [38]. Therefore, we sought to test whether the growth of HGSOC cells that show increased levels of Glu could be inhibited by an antagonist of NMDAR. A panel of HGSOC cells consisting of Kuramochi, SNU119, and TYKNU cells were treated with MK801, a potent antagonist of NMDAR [39]. As shown in Figure 9, the inhibitor attenuated the proliferation of these cells in a dependent manner.

### 3.7. Evaluating 5-HT-Mediated Autocrine Signaling in HGSOC Cells

**Figure 10:**
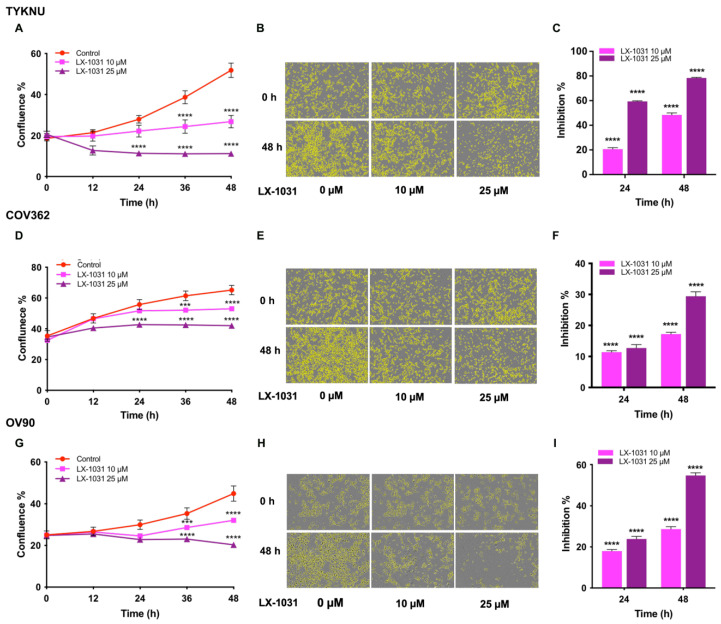
Inhibition of Ovarian Cancer Cell Proliferation by LX-1031, an antagonist of 5-HT synthesis. TYKNU, COV362 and OV90, cells were treated with vehicle control or LX-1031at concentrations of 10 µM and 25 µM. Cell proliferation was monitored for 48 h in IncuCyte S3 Live-Cell Analysis system as detailed under Materials and Methods. (**A**,**D**,**G**). Cell proliferation are expressed in terms of total confluence % of the cells; (**B**,**E**,**H**). Phase contrast bright field micrographs of cells with cell masks (yellow) at 10× magnification are presented. (**C**,**F**,**I**) Percent inhibitions over the untreated control are presented. Results are from a typical experiment (n = 3) and the error bars represent mean ± SEM. Statistical significance, determined by two-way ANOVA and Dunnett’s multiple comparison post-hoc test, are denoted by ***, *p* < 0.0005; ****, *p* < 0.0001.

Similar to Glu and GABA receptors, 5-HT or serotonin receptors (5-HT-R) show extensive heterogeneity and at least four different isoforms of the receptor have been reported to be expressed in ovarian cancer [40,41]. There is some evidence that methiothepin, a pan-antagonist of 5-HT-R could inhibit the growth of HGSOC cell lines, ES2 and OV90 [39]. However, the contributory role of cancer cell-derived 5-HT in triggering an oncogenic autocrine signaling loop in ovarian cancer has not been defined yet. We tested this using LX-1031, an inhibitor of tryptophan hydroxylase 1, which has been widely used to inhibit the synthesis of 5-HT [42,43]. HGSOC cells that showed higher levels of 5-HT, namely, TYKNU, COV362, and OV90, were treated with the two different doses of LX-1031. Results indicated that the inhibition of 5-HT synthesis by LX301 attenuated the proliferation of the HGSOC cell lines in a dose-dependent manner (Figure 10).

### 3.8. Inhibition of Ovarian Cancer Cell Spheroid Growth by GluR-, GABA-R, and 5-HT Antagonists

**Figure 11:**
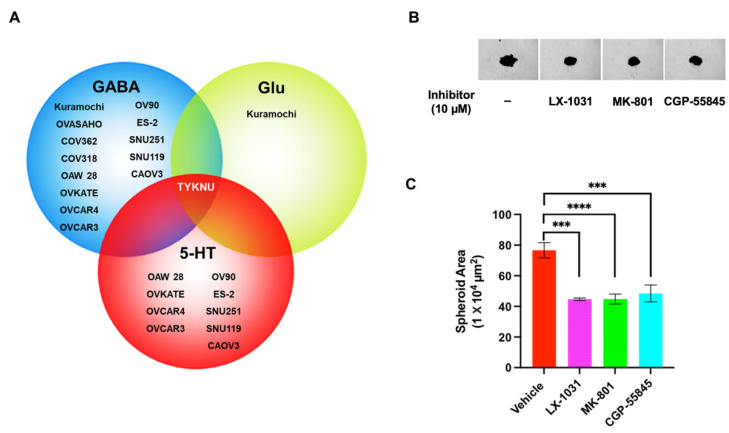
LX-1031, MK-801, and CGP-55845 treatment inhibits spheroid formation in ovarian cancer cell lines. (**A**) TYKNU cell line, which was identified as the single cell line that showed increased levels of all the three oncometabolites, was opted for the 3D spheroid assay. TYKNU cells were culture as 3D spheroids as described under methods. Effect of vehicle control and inhibitors on spheroid formation was monitored for 6 days in IncuCyte S3 Live-Cell Analysis system; (**B**) Phase contrast micrographs of each well at 4× magnification (144 h). Vehicle (DMSO) control is denoted by “–”; (**C**) Spheroid growth was quantified by quantifying the total spheroid area (µm^2^). Results from a representative experiment is presented (n = 3; Mean ± SEM). Statistical significance was determined by Student’s t-test (***, *p* < 0.0005; ****, *p* < 0.0001).

Cells grown as three-dimensional spheroids have been shown to mimic *in vivo* cellular responses to a variety of therapeutic agents [44,45]. Therefore, we tested the efficacy of these pharmacological agents on the 3D spheroid growth of HGSOC cells. Spheroid growth was initiated with TYKNU cells, a consensus cell line that showed increased levels of GABA, Glu, and 5-HT (Figure 11A). Treatment of spheroids with CGP-55845, MK-801, or LX-1031 potently inhibited the spheroid tumor growth (Figure 11B). Quantification of the results indicated that the spheroid growth, as represented by the area of the spheroid, was reduced by 40–50% (Figure 11C).

## 4. Discussion

Ovarian cancer is a heterogenous disease, characterized by several molecular subtypes with underlying genetic, epigenetic, and metabolic abnormalities [46,47,48,49]. A multilayered analysis, utilizing transcriptomic, proteomic, and epigenetic data, has identified nine molecular subtypes in ovarian cancer [46]. Mutational analyses have classified ovarian cancer into nine sub-groups [47]. Epigenetic analysis, specifically, DNA-methylation profiling has discerned 3–4 molecular subtypes [46,48,50]. An in silico analysis of the expression profiles of the genes involved in metabolic pathways has indicated three molecular subtypes although only one subtype, C2, is characterized by metabolism related genetic signatures [49]. In light of these extensive molecular heterogeneity in ovarian cancer, molecular profiling of cancer cells is one of the primary criteria to assess the closeness of the cells to each other and more importantly, to tumor samples. This is especially true with cancer cell lines that are being used as in vitro surrogates of tumor tissues. Utilizing genomic profiling, previous studies have contributed significantly in identifying ovarian cancer cell lines that closely resemble ovarian cancer tissues [51,52]. All of the 14 cells tested here were identified as HGSOC cells. However, metabolic fingerprinting of these cells, as presented here, has revealed the presence of five metabolic sub-types among these cell lines (Figure 1, Figure 2 and Figure 3). A case in point is that the mutational profiling has placed SNU119, OVCAR4, Kuramochi, OVSAHO, COV318, and COV362 cell-lines closer to each other than to others [51]. However, the metabolic fingerprinting, presented here, segregates this group further into three sub-groups comprised of (1) SNU119, Kuramochi, and OVSAHO; (2) COV362 and OVCAR4; and (3) COV318 (Figure 3). One of the limitations of the present study is the use of duplicate experimental samples of each cell line for the metabolomic analysis. However, the tightness of the duplicate samples in the box plots for each of the metabolites (Figure 4, Figure 5 and Figure 6) strongly supports our conclusion on metabolic clustering of the HGSOC cells. Although our results need to be strengthened by a greater number of samples, the finer classification of HGSOC cells as presented here, could be utilized for the development of effective metabolomics-based targeted therapy for ovarian cancer. At present, the genomic, epigenetic, mutational or transcriptomic basis that define the metabolic heterogeneity remains to be clarified. Further multiomics-based characterization of these cell lines should define the etiological basis metabolic diversity, resultant clustering of the cells into subgroups, and their relationship to the known molecular sub-types of ovarian cancer. Since cell lines are invariably used as surrogates in vitro tumor models, insights into the metabolic heterogeneity reported here would be of critical importance for precision cancer medicine strategies. 

Cancer metabolism has been described to be comprised of both convergent and divergent metabolic pathways [7]. It has also been surmised that metabolic convergence involves metabolic pathways underlying energy metabolism, metabolite biosynthesis, and redox regulation [7]. Our results agree with the general paradigm in which the pathways involved in energy metabolism, amino acid, and lipid biosynthesis and glutathione synthesis show overall commonality in majority of the tested HGSOC cells. However, our results also indicate specific differences in these pathways among these cell lines that can be leveraged for sub-type specific targeted therapy. One such difference is observed in sub-type specific differences in TCA cycle metabolites. Tumor cells often reduce mitochondrial oxidative phosphorylation and TCA cycle throughput and this is evident in the lower TCA cycle metabolites such as citrate and/or succinate in HGSOC cell lines, relative to the FTE188 control cells (Figure 4). Increased aerobic glycolytic activity (Warburg effect) is frequently observed in tumor cells and most of the HGSOC cell lines showed evidence of elevated glycolysis as indicated by the increase in the levels of lactate (Figure 3). It is significant to note that the changes to energy metabolism are not uniform across all cell lines. It is possible that the PTEN/PI3K/Akt pathway status play a determinant role in the energy homeostasis and resultant dependency of cancer cells to oxidative phosphorylation versus aerobic glycolysis [53]. In many instances, the PTEN/PI3K/Akt/mTOR pathway has been associated with the upregulation of oxidative phosphorylation involving TCA cycle. A recent study has shown the differential sensitivity of low-grade serous ovarian carcinoma cells and HGSOC cells to mTOR inhibitors [54]. Previous studies from several laboratories including ours have demonstrated the role of atypical signaling pathways such as those regulated by LPA and BCL20 in modulating the glucose metabolism in cancer cells [16,55,56]. Further interrogation of these signaling pathways in relation to specific metabolites should identify the mechanism(s) involved in the differential modulation of TCA cycle and aerobic glycolysis in these cell lines. Our results indicate that even within HGSOC group of cells, there is a wide variation in the metabolic pathways involving the utilization of glucose and the metabolic signature of lactate versus citrate/succinate, which can be used to determine whether the subtype of cancer cells would respond to glycolysis inhibitors [57] or TCA-cycle inhibitors [58]. 

In contrast to glucose metabolism, all the tested cell lines excepting SNU119 and ES2 showed increased phospholipid metabolism suggesting metabolic convergence in these cells (Figure 5). Phospholipids are critically involved in the pathophysiology of the cancer cells [59]. In addition to serving as the cellular energy storage depot and building blocks of cell membrane, they are also involved in oncogenic signaling, membrane fluidity, and inter cellular communications that underlie cancer growth, progression, and metastasis [60,61,62,63]. Thus, the observed increase in phospholipids correlates well with the known tumor promoting roles of phospholipids. More importantly, these findings suggest that the tested HGSOC cells share common vulnerability in phospholipid synthesis and enzyme involved in phospholipid metabolism could be targeted for therapy. This is consistent with the findings that inhibitors of general lipid metabolism could attenuate the proliferation of ovarian cancer cell lines [64,65]. Our results extend this further by demonstrating that targeting phospholipid metabolism would be a better therapeutic strategy for HGSOC cancer cells. 

Increased amino acid metabolism involving cysteine, glutamine, glutamate/glutamic acid, and tryptophan is seen in several of the tested HGSOC cell (Figure 6). Increased levels of cystathionine and cysteine along with glutathione synthesis form another integrated locus of metabolic convergence (Figure 6A–D). Although cell line to cell line variability was observed for metabolites in the glutathione synthesis pathway, all of the tested cells—excepting ES2—showed increased levels of GSH. GSH is primarily involved in the stringent regulation of redox state of the cells with its anti-oxidant activity. Increased levels of GSH contributes to therapy resistance by quenching therapy-induced reactive oxygen species involved in cell death [66,67]. Thus, our results further validate the targeting of glutathione and glutathione synthetic pathway as a therapeutic strategy in ovarian cancer. Increased amino acid metabolism involves the overexpression of proteins associated with amino acid synthesis or their uptake in many other cancers including ovarian cancer [68,69,70,71,72]. Although such overexpression of critical proteins involved in amino acid metabolism remains to be established in HGSOC cells, our findings identify amino acid metabolism as a potential therapeutic target in ovarian cancer. These results are highly significant in light of the emerging role of amino acid metabolic pathways as therapeutic targets in many cancers [70,73,74]. 

A highly significant observation is that a subset of cells showed higher levels of Glutamate, GABA, or 5-HT (Figure 6). GABA, Glu, and 5-HT have been well characterized as neurotransmitters as well as neurotrophic factors that activate intracellular signaling pathway via the stimulation of specific sets of cell surface receptors [23,24,25,75,76,77,78,79,80,81,82]. Evidence is emerging that they can elicit diverse non-neuronal response by stimulating their receptors in non-neuronal tissues and recent studies have shown that the extra-neuronal synthesis and release of neurotransmitters cells into the TME by the cancer cells promote tumorigenesis and tumor progression in many cancers [23]. While these transmitters have been shown to stimulate cell proliferation in different cancers [75,76,77,78,79,80,81,82], identification of them as potential oncometabolites or their role as mediators of autocrine signaling in ovarian cancer is hitherto unreported. Our results, presented here indicate that the antagonists of these metabolism-derived ligands can effectively disrupt the autocrine signaling loop and inhibit ovarian cancer cell proliferation (Figure 7, Figure 8, Figure 9, Figure 10 and Figure 11). In this context, it should be noted here that baclofen, a selective agonist for GABA_B_ receptor [83], has been shown to inhibit cell proliferation in SKOV3 cells [84], a cell line that represent clear cell carcinoma of the ovary [51]. Contrary to this observation, our studies indicate that the GABA_B_-antagonist inhibit cell proliferation in multiple ovarian cancer cell lines representing HGSOC (Figure 7). It is possible that GABA_B_ receptors are differentially coupled to a growth-inhibitory response in SKOV3 cells, compared to HGSOC cells. The differential effects seen between clear cell carcinoma-derived SKOV3 cells and HGSOC cells studied here, further underscores the importance of considering inter-tumoral metabolic heterogeneity in precision cancer medicine. Our results are further substantiated by the previous findings that Glutamate, GABA, and 5-HT stimulate pro-tumorigenic pathways through autocrine as well as paracrine mechanism in many different cancers. Additionally, 5-HT has been shown to be involved in oncogenic signaling and anti-tumor immunity in multiple cancers [25,26]. Similarly, GABA has been shown to promote the proliferation of pancreatic, prostate, and hepatocellular carcinoma cells [28,29]. Glutamate has also shown to stimulate the proliferation of multiple cancer cells including breast cancer through its cognate receptors [22,30]. In fact, GABA as an oncometabolite in breast [85] and prostate cancer cells [86] whereas the glutamate as an oncometabolite in thyroid cancer [87]. Oncometabolites are defined as “metabolites whose great quantity were elevated noticeably in tumors compared with normal cells” [30,31] as well as metabolites that drive “critical epigenetic changes and signaling pathway activation that affect the biological properties of cancer cells” [88]. Based on these criteria, our studies define glutamate, GABA, and 5-HT as oncometabolites that promote cell proliferation in ovarian cancer cells.

**Figure 12:**
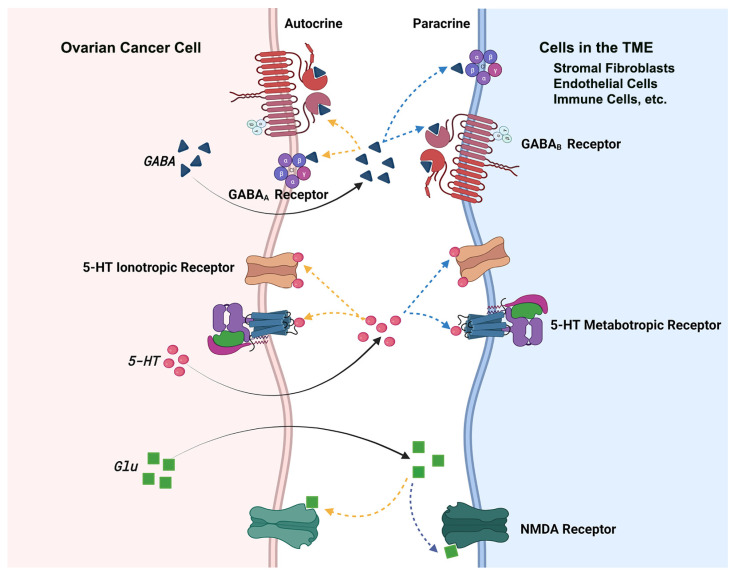
Schematic Representation of Putative Autocrine and Paracrine Signaling by GABA, Glu, and 5-HT. GABA, Glu, and 5-HT, which are synthesized within the cancer cells, are secreted into the tumor microenvironment and can bind to their specific receptors, either through autocrine or paracrine pathways in the tumor microenvironment to initiate the intracellular signaling. While autocrine signaling involves the cancer cells in which the metabolites are synthesized and released, paracrine signaling could involve the activation of tumor-promoting pathways in the TME resident stromal fibroblasts, endothelial cells and immune cells such as microglia, macrophages, and lymphocytes.

In addition to the categorizing the HGSOC cells into distinct metabolic sub-groups, our results have identified GABA, Glu, and 5-HT as oncometabolites that promote cell proliferation in ovarian cancer cells through an autocrine signaling loop (Figure 12).

The paradigm presented here also indicate the possibility that the metabolite derived from the cancer cells can also act upon other cell types in the tumor microenvironment such as cancer associated fibroblasts, endothelial cells, and immune cells to promote cancer progression. Although there is no direct evidence is presented for paracrine signaling in the present study, multiple lines of evidence from other cancers support for such a dual signaling—autocrine and paracrine—role for Glu, GABA, and 5-HT [89]. Potential role for such signaling in ovarian cancer is evidenced by the increased expression of Glu, GABA, and 5-HT receptors in ovarian cancer cells as well as tissues [38,40,90,91]. In fact, MK801 and ifenprodil, the antagonists of NMDA-family of Glu-receptor, have been shown to inhibit the proliferation of non-serous ovarian cancer cell lines SKOV3 and A2780 [38]. More interestingly, methiothepin, an antagonist of 5-HT receptors has been shown to suppress the proliferation and viability of HGSOC cell lines, ES2 and OV90 [91]. Demonstration of the paracrine signaling by fallopian tube-derived norepinephrine, yet another neurotransmitter with non-neuronal function, in ovarian cancer genesis and progression adds further support to such a dual role for Glutamate, GABA, and 5-HT in ovarian cancer progression [92]. Considering the fact that HGSOC represents the most aggressive and predominant sub-type of ovarian cancer, our results derived from HGSOC cells are highly significant in identifying metabolic subtype-specific potential therapeutic targets in ovarian cancer. Future studies comparing the metabolic fingerprints of the HGSOC cell lines with those of patient-derived cancer cells and devising methodologies for the targeted delivery of GABA, Glu, and 5-HT antagonists to ovarian cancer tissue should pave the way for novel metabolic fingerprinting-based targeted therapy in ovarian cancer. 

## Data Availability

The metabolomics dataset analyzed in the present study are deposited on 20 September 2021 and it is available at the NIH Common Fund’s National Metabolomics Data Repository (https://www.metabolomicsworkbench.org/data/DRCCMetadata.php?Mode=Project&ProjectID=PR001259; accessed on 13 January 2020).

## Supplementary Materials

The following are available online at https://www.mdpi.com/article/10.3390/biomedicines9121927/s1, Figure S1: Higher tryptophan and kynurenine levels in the subset of HGSOC cells, Table S1: Quality control samples, Table S2: Quality control Standarads, Table S3: Data Quality, Table S4: Summary of the number of metabolites identified, and Table S5. List of metabolites examined and their chemical class unique identifiers.
