# Supplementary material for: Unraveling Autocrine Signaling Pathways through Metabolic Fingerprinting in Serous Ovarian Cancer Cells
Source: Biomedicines. 2021 Dec 16;9(12):1927. doi: 10.3390/biomedicines9121927 (PMC8698993; doi:10.3390/biomedicines9121927)
Supplement: Supplementary file 1 [file biomedicines-09-01927-s001.zip › biomedicines-1431957-supplementary.pdf]

Supplementary Files

Supplemental Figure S1

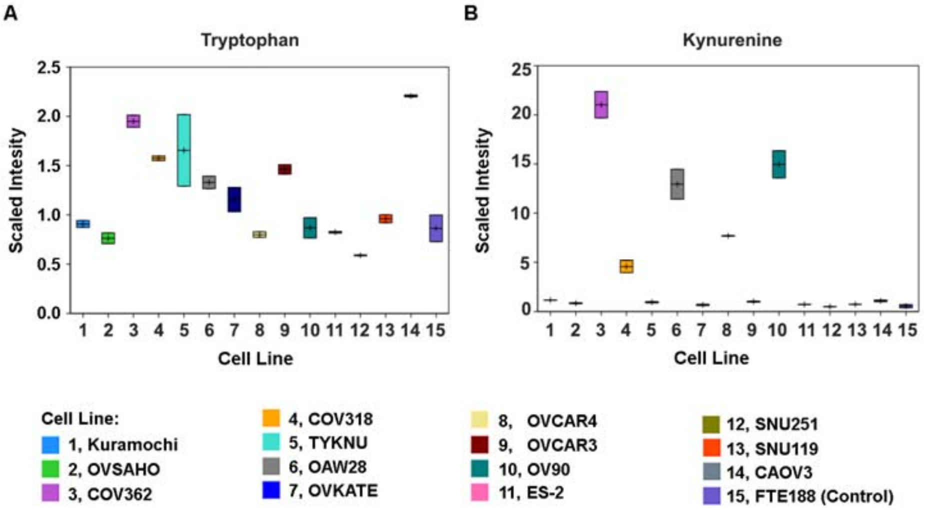

Figure S1. Higher tryptophan and kynurenine levels in the subset of HGSOC cells. Changes in the cellular content of tryptophane (A) and its metabolites Kynurenine (B) and 5-hydroxytrptamine/serotonin (Figure 4 in the text) were determined. Relative contents of the respective metabolites are expressed as scaled intensity in the Y-axis against the cell types presented in the X-axis. Center line in boxes in the box plot denotes the median value. The upper and lower borders define the two measurements of scaled intensity.

Supplemental Methods

Metabolic Analysis at Metabolon, Inc.

Sample Accession: Frozen pellets of the replicate samples of the cell lines were inventoried and immediately stored at -80°C. Each sample received was accessioned into the Metabolon LIMS system and was assigned by the LIMS a unique identifier that was associated with the original source identifier only. This identifier was used to track all sample handling, tasks, results, etc. The samples (and all derived aliquots) were tracked by the LIMS system. All portions of any sample were automatically assigned their own unique identifiers by the LIMS when a new task was created; the relationship of these samples was also tracked. All samples were maintained at -80°C until processed.

Sample Preparation: Samples were prepared using the automated MicroLab STAR® system (Hamilton Company, Reno, NV, USA). Recovery standards were added prior to the extraction process for quality control (QC) purposes. Proteins in the cell lysates were precipitated with methanol followed by centrifugation. The methanol extract was divided into five fractions: two for analysis by two separate reverse phase (RP)/UPLC-MS/MS methods with positive ion mode electrospray ionization (ESI), one for analysis by RP/UPLC-MS/MS with negative ion mode ESI, one for analysis by HILIC/UPLC-MS/MS with negative ion mode ESI, and one sample was reserved for backup. Organic solvent was evaporated and the extracted samples were stored under nitrogen prior to analysis.

Quality control (QC): Aliquots of pooled samples or a pool of well-characterized human plasma was used as technical replicate. A defined set of QC standards were spiked into every analyzed sample to monitor instrument performance and chromatographic alignment. Instrument variability was determined by calculating the median relative standard deviation (RSD) for the standards that were added to each sample prior to injection into the mass spectrometers. Tables S1 and S2 describe these QC samples and standards. Overall process variability was determined by calculating the median RSD for all endogenous metabolites present in 100% of the pooled matrix samples.

Table S1. Quality Control Samples

Type	Description	Purpose
MTRX	Large pool of human plasma maintained by Metabolon that has been characterized extensively.	Assure that all aspects of the Metabolon process are operating within specifications.
CMTRX	Pool created by taking a small aliquot from every customer sample.	Assess the effect of a non-plasma matrix on the Metabolon process and distinguish biological variability from process variability.
PRCS	Aliquot of ultra-pure water	Process Blank used to assess the contribution to compound signals from the process.
SOLV	Aliquot of solvents used in extraction.	Solvent Blank used to segregate contamination sources in the extraction.

Table S2. Quality Control Standards

Type	Description	Purpose
RS	Recovery Standard	Assess variability and verify performance of extraction and instrumentation.
IS	Internal Standard	Assess variability and performance of instrument.

Data Quality: Instrument and Process Variability: Instrument variability was determined by calculating the median relative standard deviation (RSD) for the internal standards that were added to each sample prior to injection into the mass spectrometers. Overall process variability was determined by calculating

the median RSD for all endogenous metabolites (i.e., non-instrument standards) present in 100% of the Client Matrix samples, which are technical replicates of pooled client samples. Values for instrument and process variability meet Metabolon's acceptance criteria as shown in the table S3.

Table S3. Data Quality

QC Sample	*Measurement*	*Median RSD*
**Internal Standards**	**Instrument Variability**	**5%**
**Endogenous Biochemicals**	**Total Process Variability**	**7%**

Ultrahigh Performance Liquid Chromatography-Tandem Mass Spectroscopy (UPLC-MS/MS): Waters ACQUITY ultra-performance liquid chromatography (UPLC) and a Thermo Scientific Q-Exactive high resolution/accurate mass spectrometer interfaced with a heated electrospray ionization (HESI-II) source and Orbitrap mass analyzer operated at 35,000 mass resolution were used. Sample extracts were dried, then reconstituted in solvents compatible to each of the four methods as follows. Each reconstitution solvent contained a series of standards at fixed concentrations to ensure injection and chromatographic consistency. One aliquot was analyzed using acidic positive ion conditions, chromatographically optimized for more hydrophilic compounds. In this method, the extract was gradient eluted from a C18 column (Waters UPLC BEH C18-2.1x100 mm, 1.7 µm) using water and methanol, containing 0.05% perfluoropentanoic acid (PFPA) and 0.1% formic acid (FA). Another aliquot was also analyzed using acidic positive ion conditions; however, it was chromatographically optimized for more hydrophobic compounds. In this method, the extract was gradient eluted from the same afore mentioned C18 column using methanol, acetonitrile, water, 0.05% PFPA and 0.01% FA and was operated at an overall higher organic content. Another aliquot was analyzed using basic negative ion optimized conditions using a separate dedicated C18 column. The basic extracts were gradient eluted from the column using methanol and water, however with 6.5mM Ammonium Bicarbonate at pH 8. The fourth aliquot was analyzed via negative ionization following elution from a HILIC column (Waters UPLC BEH Amide 2.1x150 mm, 1.7 µm) using a gradient consisting of water and acetonitrile with 10mM Ammonium Formate, pH 10.8. The MS analysis alternated between MS and data-dependent MS^n scans using dynamic exclusion. The scan range varied between methods but covered 70-1000 m/z. Raw data files were archived and extracted for further analysis.

Bioinformatics: The informatics system consisted of four major components, the Laboratory Information Management System (LIMS), the data extraction and peak-identification software, data processing tools for QC and compound identification, and a collection of information interpretation and visualization tools for use by data analysts. The hardware and software foundations for these informatics components were the LAN backbone, and a database server running Oracle 10.2.0.1 Enterprise Edition.

LIMS: The purpose of the Metabolon LIMS system was to enable fully auditable laboratory automation through a secure, easy to use, and highly specialized system. The scope of the Metabolon LIMS system encompasses sample accessioning, sample preparation and instrumental analysis and reporting and advanced data analysis. All of the subsequent software systems are grounded in the LIMS data structures. It has been modified to leverage and interface with the in-house information extraction and data visualization systems, as well as third party instrumentation and data analysis software.

Data Extraction and Compound Identification: Raw data was extracted, peak-identified and QC processed using Metabolon's hardware and software. Compounds were identified by comparison to library entries of purified standards or recurrent unknown entities. Metabolon maintains a library based on authenticated standards that contains the retention time/index (RI), mass to charge ratio (*m/z)*, and

chromatographic data (including MS/MS spectral data) on all molecules present in the library. Furthermore, biochemical identifications are based on three criteria: retention index within a narrow RI window of the proposed identification, accurate mass match to the library +/- 10 ppm, and the MS/MS forward and reverse scores between the experimental data and authentic standards. The MS/MS scores are based on a comparison of the ions present in the experimental spectrum to the ions present in the library spectrum. While there may be similarities between these molecules based on one of these factors, the use of all three data points can be utilized to distinguish and differentiate biochemicals. More than 3300 commercially available purified standard compounds have been acquired and registered into LIMS for analysis on all platforms for determination of their analytical characteristics. Additional mass spectral entries have been created for structurally unnamed biochemicals, which have been identified by virtue of their recurrent nature (both chromatographic and mass spectral). These compounds have the potential to be identified by future acquisition of a matching purified standard or by classical structural analysis.

Curation: A variety of curation procedures were carried out to ensure that a high-quality data set was made available for statistical analysis and data interpretation. The QC and curation processes were designed to ensure accurate and consistent identification of true chemical entities, and to remove those representing system artifacts, mis-assignments, and background noise. Metabolon data analysts use proprietary visualization and interpretation software to confirm the consistency of peak identification among the various samples. Library matches for each compound were checked for each sample and corrected if necessary.

Metabolite Quantification and Data Normalization: Peaks were quantified using area-under-the-curve. For studies spanning multiple days, a data normalization step was performed to correct variation resulting from instrument inter-day tuning differences. Essentially, each compound was corrected in run-day blocks by registering the medians to equal one (1.00) and normalizing each data point proportionately (termed the "block correction). For studies that did not require more than one day of analysis, no normalization is necessary, other than for purposes of data visualization. Biochemical data were normalized to total protein as determined by Bradford assay to account for differences in metabolite levels due to differences in the amount of material present in each sample.

Statistical Calculations: For many studies, two types of statistical analysis are usually performed: (1) significance tests and (2) classification analysis. Standard statistical analyses were performed in ArrayStudio 7.2 (Qiagen OmicSoft, Cary, NC) on log transformed data to compare the data from the experimental groups using Multivariate ANOVA. For those analyses not standard in ArrayStudio, the programs R (http://cran.r-project.org/) or JMP JMP software (JMP Inc. Cary, NC) were used. Post-hoc P-values and false discovery rates were calculated by determining the p- and q-values using Storey's method (Storey, J.D.; Tibshirani, R. Statistical significance for genomewide studies. *Proc Natl Acad Sci U S A* **2003**, *100*, 9440-9445, doi:10.1073/pnas.1530509100). Only the data with the p-value of < 0.05 were considered significant. FDR values of < 0.05 were considered significant. Complete dataset with p- and q-values are deposited at the NIH Common Fund's National Metabolomics Data Repository:

(https://www.metabolomicsworkbench.org/data/DRCCMetadata.php?Mode=Project&ProjectID=PR001259).

Hierarchical Clustering: Unsupervised Hierarchical clustering method was used for clustering the data. and can show large-scale differences. Clustering used the Euclidean distance, where each sample is a vector with all of the metabolite values.

Principal Components Analysis (PCA). Unsupervised PCA was carried out to reduces the dimension of the data. Each principal component is a linear combination of every metabolite and the principal components are uncorrelated. The number of principal components is equal to the number of observations. In 2D-PCA, the first principal component is computed by determining the coefficients of the metabolites

that maximizes the variance of the linear combination. The second component finds the coefficients that maximize the variance with the condition that the second component is orthogonal to the first. In 3D-PCA, along with the above-mentioned components, the third component, which is orthogonal to the first two components is included. The total variance is defined as the sum of the variances of the predicted values of each component, and for each component, the proportion of the total variance is computed. The total variance is defined as the sum of the variances of the predicted values of each component), and for each component, the proportion of the total variance is computed.

Experimental Summary

Replicate cell pellet samples representing fourteen ovarian cancer lines and control cell line were collected and analyzed for global metabolic profiling. Global metabolic profiles were determined using Metabolon metabolomic analysis platform. Following normalization to Bradford protein concentration, log transformation and imputation of missing values, if any, with the minimum observed value for each compound, Welch's two-sample *t*-test was used to identify biochemicals that differed significantly between experimental groups. A summary of the numbers of biochemicals that achieved statistical significance ($p \leq 0.05$) is presented in Table 4. An estimate of the false discovery rate (*q*-value) is calculated to take into account the multiple comparisons that normally occur in metabolomic-based studies. For example, when analyzing 200 compounds, one would expect to see about 10 compounds meeting the $p \leq 0.05$ cut-off by random chance. The *q*-value describes the false discovery rate; a low *q*-value ($q<0.0.05$) is an indication of high confidence in a result. The present dataset comprises a total of 731 compounds of known identity (Table 5). Complete set of data along with the p- and q-values are deposited at the NIH Common Fund's National Metabolomics Data Repository:

(https://www.metabolomicsworkbench.org/data/DRCCMetadata.php?Mode=Project&ProjectID=PR001259)

Data Presentation

Relative contents of the respective metabolites are expressed as scaled intensity in the Y-axis against the cell types presented in the X-axis. Scaled intensity is an arbitrary unit relative to the overall median 1 for the test metabolite. Raw intensity data of each metabolite was scaled on the intensity of the commercially available biochemical metabolite analyzed using the same instrument. Data points are presented as boxes in the box plot in which the center line in boxes in denotes the median value. The upper and lower borders define the 25th and 75th quartiles of scaled intensity.

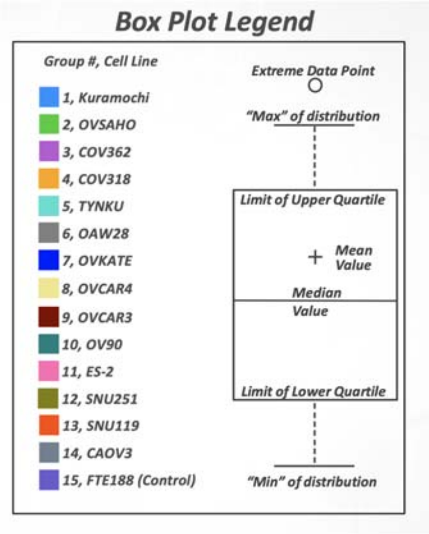

Table S4: Summary of the number of metabolites identified. Statistical analyses were performed in ArrayStudio version 7.2 (Qiagen OmicSoft, Cary, NC) on log transformed data to compare the data from the experimental groups using Multivariate ANOVA. Post-hoc P-values and false discovery rates were calculated by determining the p- and q-values using Storey's method.

**Cell Lines**		**Metabolites p<0.05**		
**Ovarian cancer cell line**	**Control**	**Total**	**Upregulated**	**Downregulated**
Kuramochi	FTE188	179	114	65
OVSAHO	FTE188	155	104	51
COV362	FTE188	157	78	79
COV318	FTE188	141	74	67
TYNKU	FTE188	160	81	79
OAW28	FTE188	209	149	60
OVKATE	FTE188	154	101	23
OVCAR4	FTE188	173	66	107
OVCAR3	FTE188	194	143	51
OV90	FTE188	152	107	45
ES-2	FTE188	222	114	108
SNU251	FTE188	150	64	86
SNU119	FTE188	146	82	64
CAOV3	FTE188	171	115	56
Sum of all groups	FTE188	289	189	100

Table S5. List of metabolites examined and their chemical class unique identifiers

Biochemical Name	Platform	Comp ID	KEGG	HMDB	PUBCHEM	Chemical ID	CAS	RI	Mass
glycine	LC/MS pos early	58	C00037	HMDB00123	750	340	56-40-6	1375	76.03931
N-acetylglycine	LC/MS pos early	27710	_	HMDB00532	10972	100001006	543-24-8	826	118.04987
sarcosine	LC/MS pos early	1516	C00213	HMDB00271	1088	1023	107-97-1	1280	90.05496
dimethylglycine	LC/MS pos early	5086	C01026	HMDB00092	673	806	1118-68-9	1104	104.07061
betaine	LC/MS pos early	3141	C00719	HMDB00043	247	799	107-43-7	1064	118.08626
betaine aldehyde	LC/MS pos early	15499	C00576	HMDB01252	249	1219	7758-31-8	1915	120.10191
serine	LC/MS pos early	1648	C00065	HMDB00187	5951	503	56-45-1	1239	106.04987
N-acetylserine	LC/MS pos early	37076	_	HMDB02931	65249	100001851	97-14-3	811	148.06044
threonine	LC/MS pos early	1284	C00188	HMDB00167	6288	564	72-19-5	1514	120.06552
N-acetylthreonine	LC/MS neg	33939	_	_	152204	100001274	17093-74-2	821.6	160.06153
allo-threonine	LC/MS polar	15142	C05519	HMDB04041	99289	100000272	28954-12-3	2511.1	118.05096
phosphothreonine	LC/MS pos early	37453	C12147	_	1016	100002076	27530-80-9	605	200.03185
alanine	LC/MS pos early	1126	C00041	HMDB00161	5950	811	56-41-7	1700	90.05496
N-acetylalanine	LC/MS neg	1585	C02847	HMDB00766	88064	1110	97-69-8	861.2	130.05096
aspartate	LC/MS pos early	443	C00049	HMDB00191	5960	234	56-84-8	1165	134.04479
asparagine	LC/MS pos early	512	C00152	HMDB00168	6267	917	70-47-3	1225	133.06077
N-acetylasparagine	LC/MS pos early	33942	_	HMDB06028	99715	100001257	4033-40-3	785	175.07134
N-acetylaspartate (NAA)	LC/MS polar	22185	C01042	HMDB00812	65065	100000787	997-55-7;997-55-7	3143	174.04079
3-sulfo-L-alanine	LC/MS polar	47089	C00506	HMDB02757	72886	382	498-40-8	2890	167.99722
glutamate	LC/MS pos early	57	C00025	HMDB00148	611	561	56-86-0	1500	148.06044
glutamine	LC/MS pos early	53	C00064	HMDB00641	5961	563	56-85-9	1291	147.07642
N-acetylglutamate	LC/MS pos early	15720	C00624	HMDB01138	70914	100000282	8/3/17	1050	190.071
N-acetylglutamine	LC/MS pos early	33943	C02716	HMDB06029	182230	100001253	2490-97-3	845	189.08699
N-acetyl-aspartyl-glutamate (NAAG)	LC/MS neg	35665	C12270	HMDB01067	5255	100001612	3106-85-2	575.3	303.08339
gamma-aminobutyrate (GABA)	LC/MS pos early	1416	C00334	HMDB00112	119	141	56-12-2	2048	104.07061
carboxyethyl-GABA	LC/MS pos early	40007	_	HMDB02201	2572	100003260	3/2/86	2085	176.09174
4-hydroxyglutamate	LC/MS pos early	40499	C03079	HMDB01344	439902	100002544	2485-33-8	1141	164.05535
glutamate, gamma-methyl ester	LC/MS pos early	33487	_	_	68662	100001103	1499-55-4	2170	162.07609
pyroglutamine*	LC/MS pos early	46225	_	_	134508	100001540	2353-44-8	1900	129.06586
beta-citrylglutamate	LC/MS neg	54923	_	_	_	100003271	73590-26-8	530	320.06232
N-methyl-GABA	LC/MS pos early	39577	_	_	1550042;70703	100003152	1119-48-8	2109	118.08626
S-1-pyrroline-5-carboxylate	LC/MS pos early	42370	C04322	HMDB01301	1196	35	2906-39-0	1528	114.05496
histidine	LC/MS neg	59	C00135	HMDB00177	6274	355	5934-29-2	755.9	154.0622
N-acetylhistidine	LC/MS pos early	33946	C02997	HMDB32055	75619	100001293	39145-52-3	2065	198.08732
1-methylhistidine	LC/MS pos early	30460	C01152	HMDB00001	92105	100001051	332-80-9	2755	170.09241
3-methylhistidine	LC/MS neg	15677	C01152	HMDB00479	64969	100000042	368-16-1	906.3	168.07785
imidazole propionate	LC/MS pos early	40730	_	HMDB02271	70630	100003434	1074-59-5	2263	141.06586

imidazole lactate	LC/MS pos early	15716	C05568	HMDB02320	440129	100000263	14403-45-3	2040	157.06077
1-methylimidazoleacetate	LC/MS pos early	32350	C05828	HMDB02820	75810	100001208	2625-49-2	2064	141.06586
4-imidazoleacetate	LC/MS pos early	32349	C02835	HMDB02024	96215	100001207	645-65-8	2055	127.05021
histidine methyl ester	LC/MS pos early	32493	_	_	92893	100001124	1499-46-3	3080	170.09241
lysine	LC/MS pos early	1301	C00047	HMDB00182	5962	407	56-87-1	2850	147.11281
N6-acetyllysine	LC/MS polar	36752	C02727	HMDB00206	92832	100001734	692-04-6	2612.6	187.10881
N6,N6,N6-trimethyllysine	LC/MS pos early	1498	C03793	HMDB01325	440120	189	23284-33-5	2825	189.15976
5-hydroxylysine	LC/MS pos early	15685	C16741	HMDB00450	1029	100000054	13204-98-3	2790	163.10772
saccharopine	LC/MS pos early	1495	C00449	HMDB00279	160556	392	997-68-2	2530	277.13942
2-aminoadipate	LC/MS pos early	6146	C00956	HMDB00510	469	381	542-32-5;1118-90-7	1912	162.07609
glutarate (pentanedioate)	LC/MS polar	396	C00489	HMDB00661	743	339	110-94-1	2900	131.03498
glutarylcarnitine (C5-DC)	LC/MS pos early	44664	_	HMDB13130	71464488	100001593	102636-82-8	2393	276.14417
pipecolate	LC/MS pos early	1444	C00408	HMDB00070	849	1025	4043-87-2	2200	130.08626
N-acetyl-cadaverine	LC/MS pos early	43530	_	_	_	100002249	32343-73-0	2385	145.13354
5-aminovalerate	LC/MS pos early	18319	C00431	HMDB03355	138	100000454	660-88-8	2220	118.08626
5-(galactosylhydroxy)-L-lysine	LC/MS pos early	43582	_	_	_	100002462	32448-36-5	2755	325.16055
6-oxopiperidine-2-carboxylate	LC/MS pos early	43231	_	_	3014237	100004499	34622-39-4	1432	144.06552
phenylalanine	LC/MS pos early	64	C00079	HMDB00159	6140	460	63-91-2	2878	166.08626
N-acetylphenylalanine	LC/MS neg	33950	C03519	HMDB00512	74839	100001256	2018-61-3	2597	206.08226
phenyllactate (PLA)	LC/MS polar	22130	C05607	HMDB00779	3848	100000774	828-01-3	908	165.05571
tyrosine	LC/MS pos early	1299	C00082	HMDB00158	6057	815	60-18-4	2430	182.08117
N-acetyltyrosine	LC/MS neg	32390	_	HMDB00866	68310	100001104	537-55-3	1680	222.07718
4-hydroxyphenylpyruvate	LC/MS neg	1669	C01179	HMDB00707	979	1141	156-39-8	1690.1	179.03498
3-(4-hydroxyphenyl)lactate	LC/MS neg	32197	C03672	HMDB00755	9378	240	6482-98-0	1379	181.05063
phenol sulfate	LC/MS neg	32553	C02180	HMDB60015	74426	100001510	937-34-8	2156	172.9914
p-cresol sulfate	LC/MS neg	36103	C01468	HMDB11635	4615423	100001315	3233-57-7	2890	187.00705
o-cresol sulfate	LC/MS neg	36845	_	_	11615528	100001806	_	2796	187.00705
3-methoxytyrosine	LC/MS pos early	12017	_	HMDB01434	1670	1342	300-48-1	2555	212.09174
O-methyltyrosine	LC/MS pos early	37451	_	_	76957	100002078	1080-06-4	2790	196.09682
N-formylphenylalanine	LC/MS neg	48433	_	_	759256	100006056	13200-85-6	2360	192.06661
tryptophan	LC/MS pos early	54	C00078	HMDB00929	6305	565	73-22-3	2986	205.09716
tryptamine	LC/MS pos early	6104	C00398	HMDB00303	1150	1095	61-54-1	3062	161.10733
indolelactate	LC/MS neg	18349	C02043	HMDB00671	92904	100000463	832-97-3	2286	204.06661
kynurenine	LC/MS pos early	15140	C00328	HMDB00684	161166	100000265	2922-83-0	2739	209.09207
kynurenate	LC/MS neg	1417	C01717	HMDB00715	3845	98	492-27-3	2224	188.03531
3-hydroxykynurenine	LC/MS neg	22110	C02794	HMDB00732	89	100000986	2147-61-7	1349	223.07243
5-hydroxyindoleacetate	LC/MS neg	437	C05635	HMDB00763	1826	71	54-16-0	1510.2	190.05096
serotonin	LC/MS pos early	2342	C00780	HMDB00259	5202	504	153-98-0	2550	177.10224
tryptophan betaine	LC/MS pos early	37097	C09213	HMDB61115	442106	100001743	20671-76-5	2673	247.14411
C-glycosyltryptophan	LC/MS neg	48782	_	_	10981970	100006379	180509-18-6	1777	365.13542
thioproline	LC/MS pos early	53231	_	_	93176;6973609	100009232	34592-47-4	1250	134.02703

leucine	LC/MS pos early	60	C00123	HMDB00687	6106	397	61-90-5	2864	132.10191
N-acetylleucine	LC/MS neg	1587	C02710	HMDB11756	70912	1082	1188-21-2	2400	172.09791
4-methyl-2-oxopentanoate	LC/MS neg	22116	C00233	HMDB00695	70	100000551	816-66-0	2170	129.05571
isovaleryl/2-methylbutyryl CoA	LC/MS neg	57386	-	-	-	100015649	-	2861	850.16544
isovalerylglycine	LC/MS neg	35107	-	HMDB00678	546304	100001452	16284-60-9	1950	158.08226
isovalerylcarnitine (C5)	LC/MS pos early	34407	-	HMDB00688	6426851	100001393	31023-24-2	3085	246.16999
beta-hydroxyisovalerate	LC/MS polar	12129	-	HMDB00754	69362	1442	625-08-1	1149.8	117.05572
3-methylglutaconate	LC/MS polar	38667	-	HMDB00522	1551553	100002458	5746-90-7	2579.6	143.03498
alpha-hydroxyisovalerate	LC/MS polar	46537	-	HMDB00407	99823	100001300	600-37-3	1052	117.05571
methylsuccinate	LC/MS polar	15745	-	HMDB01844	10349	2051	498-21-5	2800	131.03498
isoleucine	LC/MS pos early	1125	C00407	HMDB00172	6306	376	73-32-5	2800	132.10191
N-acetylisoleucine	LC/MS neg	33967	-	-	2802421	100001276	3077-46-1	2325	172.09791
3-methyl-2-oxobutyrate	LC/MS polar	44526	C00141	HMDB00019	49	100000936	3715-29-5	911	115.04006
butyryl/isobutyryl CoA	LC/MS neg	57383	-	-	-	100015648	-	2375	836.14979
3-methyl-2-oxovalerate	LC/MS neg	15676	C00671	HMDB03736	47	100000036	1460-34-0;51829-07-3	2064.2	129.05572
2-methylbutyrylcarnitine (C5)	LC/MS pos early	45095	-	HMDB00378	6426901	100001509	31023-25-3	3035	246.16999
2-methylbutyrylglycine	LC/MS pos early	31928	-	HMDB00339	193872	100001155	52320-67-9	2000	160.09682
tiglylcarnitine (C5:1-DC)	LC/MS pos early	35428	-	HMDB02366	22833596	100001597	64191-86-2	2962	244.15434
2-hydroxy-3-methylvalerate	LC/MS neg	36746	-	HMDB00317	164623	100001541	488-15-3	1800	131.07136
3-hydroxy-2-ethylpropionate	LC/MS polar	32397	-	HMDB00396	188979	100001170	4374-62-3	1443.3	117.05571
ethylmalonate	LC/MS polar	15765	-	HMDB00622	11756	2054	601-75-2	2785	131.03498
valine	LC/MS pos early	1649	C00183	HMDB00883	6287	566	72-18-4	2479	118.08626
N-acetylvaline	LC/MS neg	1591	-	HMDB11757	66789	1084	96-81-1	1704	158.08226
isobutyrylcarnitine (C4)	LC/MS pos early	33441	-	HMDB00736	168379	100001055	25518-49-4	2810	232.15434
3-hydroxyisobutyrate	LC/MS polar	1549	C06001	HMDB00336	87	111	2068-83-9	1619	103.04007
alpha-hydroxyisocaproate	LC/MS neg	22132	C03264	HMDB00746	83697	100000706	10303-64-7	1840	131.07136
methionine	LC/MS pos early	1302	C00073	HMDB00696	6137	415	63-68-3	2526	150.05833
N-acetylmethionine	LC/MS neg	1589	C02712	HMDB11745	448580	1083	65-82-7	1787	190.05434
N-formylmethionine	LC/MS neg	2829	C03145	HMDB01015	439750	194	4289-98-9	1543.8	176.03869
S-methylmethionine	LC/MS pos early	38127	C05319	-	458	100002183	12/7/93	2584	164.07398
methionine sulfone	LC/MS pos early	44878	-	-	69961	100004635	820-10-0	1250	182.04816
methionine sulfoxide	LC/MS pos early	18374	C02989	HMDB02005	158980	100000039	3226-65-1	1272	166.05325
N-acetylmethionine sulfoxide	LC/MS pos early	45428	-	-	193368	100005463	108646-71-5	880	208.06381
S-adenosylmethionine (SAM)	LC/MS pos early	15915	C00019	HMDB01185	34755	1263	24346-00-7;86867-01-8;86867-0108	3002	399.14452
S-adenosylhomocysteine (SAH)	LC/MS neg	42382	C00021	HMDB00939	439155	197	979-92-0	1832.4	383.11431
homocysteine	LC/MS pos early	15128	C00155	HMDB00742	778	311	454-29-5	2155	136.04268
cystathionine	LC/MS pos early	15705	C02291	HMDB00099	439258	310	535-34-2	2270	223.07471
2-aminobutyrate	LC/MS pos early	42374	C02261	HMDB00650	439691	1128	1492-24-6	2059	104.07061
cysteine	LC/MS pos early	1868	C00097	HMDB00574	5862	800	52-90-4;56-89-3	1488	122.02703
N-acetylcysteine	LC/MS neg	1586	C06809	HMDB01890	12035	1107	616-91-1	1108	162.02304

cystine	LC/MS polar	56	C00491	HMDB00192	67678	279	56-89-3	3424	239.01657
cysteine sulfinic acid	LC/MS pos early	37443	C00606	HMDB00996	109	100002113	207121-48-0	597	154.01686
hypotaurine	LC/MS pos early	590	C00519	HMDB00965	107812	358	300-84-5	724	110.02703
taurine	LC/MS neg	2125	C00245	HMDB00251	1123	512	107-35-7	690	124.00739
N-acetyltaurine	LC/MS polar	48187			159864	100005466		1249	166.01795
2-hydroxybutyrate/2-hydroxyisobutyrate	LC/MS polar	52281				100008928		1258	103.04006
arginine	LC/MS pos early	1638	C00062	HMDB00517	232	231	1119-34-2	2825	175.11896
ornithine	LC/MS pos early	1493	C00077	HMDB03374	6262	444	3184-13-2	2800	133.09716
proline	LC/MS pos early	1898	C00148	HMDB00162	145742	480	147-85-3	1603	116.07061
citrulline	LC/MS pos early	2132	C00327	HMDB00904	9750	391	372-75-8	1520	176.10297
argininosuccinate	LC/MS pos early	15497	C03406	HMDB00052	16950;828	232	156637-58-0	2745	291.12992
homoarginine	LC/MS pos early	22137	C01924	HMDB00670	9085	100000961	156-86-5	2882	189.13461
homocitrulline	LC/MS pos early	22138	C02427	HMDB00679	65072	100000963	1190-49-4	1908	190.11862
dimethylarginine (SDMA + ADMA)	LC/MS pos early	36808	C03626	HMDB01539	123831	100001810	102783-24-4	2850	203.15026
N-acetylarginine	LC/MS pos early	33953	C02562	HMDB04620	67427	100001266	155-84-0	2245	217.12952
N-delta-acetylornithine	LC/MS pos early	43249			9920500	100004523		1877	175.10772
N-methylproline	LC/MS pos early	37431			557	100001956	475-11-6	1335	130.08626
trans-4-hydroxyproline	LC/MS pos early	32306	C01157	HMDB00725	5810	1001	51-35-4	1064	132.06552
pro-hydroxy-pro	LC/MS pos early	35127		HMDB06695	11673055	100001167	18684-24-7	2128	229.11829
N-monomethylarginine	LC/MS pos early	43586	C03884	HMDB29416	132862	100004488	53308-83-1	2845	189.13461
2-oxoarginine*	LC/MS neg	55072	C03771	HMDB04225	558	100002784	10/4/15	963	172.07276
argininate*	LC/MS pos early	57461		HMDB03148	160437	100002769	157-07-3	2150	176.10297
creatine	LC/MS pos early	27718	C00300	HMDB00064	586	1221	57-00-1	1947	132.07676
creatinine	LC/MS pos early	513	C00791	HMDB00562	588	275	60-27-5	2055	114.06619
creatine phosphate	LC/MS polar	33951	C02305	HMDB01511	587	100000112	922-32-7	3831	210.02853
guanidinoacetate	LC/MS pos early	43802	C00581	HMDB00128	763	344	352-97-6	1937	118.06111
putrescine	LC/MS pos early	1408	C00134	HMDB01414	1045	49	110-60-1	3027	89.10733
N1,N12-diacetylspermine	LC/MS pos early	52987	C03413	HMDB02172	132680	100002132	61345-83-3	3105	287.24416
spermidine	LC/MS pos early	485	C00315	HMDB01257	1102	50	124-20-9	3355	146.16518
5-methylthioadenosine (MTA)	LC/MS pos early	1419	C00170	HMDB01173	439176	212	2457-80-9	2752	298.09684
N(1)-acetylspermine	LC/MS pos early	32360	C02567	HMDB01186	916	1200	77928-70-2	3356	245.23359
N(4)-acetylspermidine	LC/MS pos early	32356			128317	100001214	66039-56-3	3095	188.17574
N-acetylputrescine	LC/MS pos early	37496	C02714	HMDB02064	122356	192	18233-70-0	2230	131.11789
4-acetamidobutanoate	LC/MS pos early	1558	C02946	HMDB03681	18189	1113	3025-96-5	1350	146.08117
4-guanidinobutanoate	LC/MS pos early	15681	C01035	HMDB03464	500	100000096	463-003;463-00-3	2320	146.09241
glutathione, reduced (GSH)	LC/MS pos early	2127	C00051	HMDB00125	124886	496	70-18-8	2000	308.09109
glutathione, oxidized (GSSG)	LC/MS pos early	27727	C00127	HMDB03337	65359	448	103239-24-3	2667	613.15925
cysteine-glutathione disulfide	LC/MS pos early	35159		HMDB00656	4247235	100001437	13081-14-6	2465	427.09519
S-methylglutathione	LC/MS neg	33944	C11347		3605667	100001259	2922-56-7	1075	320.09218
S-lactoylglutathione	LC/MS neg	15731	C03451	HMDB01066	440018	1343	54398-03-7	1248	378.09766
cysteinylglycine	LC/MS pos early	35637	C01419	HMDB00078	439498	278	19246-18-5	2132	179.0485

5-oxoproline	LC/MS neg	1494	C01879	HMDB00267	7405	1021	98-79-3	738.5	128.03531
ophthalmate	LC/MS pos early	34592	_	HMDB05765	7018721	100001311	495-27-2	2085	290.13467
S-nitrosoglutathione (GSNO)	LC/MS pos early	47127	_	HMDB04645	3514	100004662	57564-91-7	2460	337.08125
4-hydroxy-nonenal-glutathione	LC/MS neg	48504	_	_	_	100006240	99927-70-5	3387.5	462.19156
gamma-glutamylalanine	LC/MS pos early	37063	_	HMDB29142	440103	100001843	5875-41-2	1986	219.09755
gamma-glutamylcysteine	LC/MS pos early	1778	C00669	HMDB01049	842	1036	636-58-8	2118	251.06963
gamma-glutamylglutamate	LC/MS pos early	36738	C05282	HMDB11737	92865	331	1116-22-9	1775	277.10303
gamma-glutamylglutamine	LC/MS pos early	2730	C05283	HMDB11738	150914	1140	10148-81-9	1430	276.11902
gamma-glutamylglycine	LC/MS pos early	33949	_	HMDB11667	165527	100001294	1948-29-4	1535	205.0819
gamma-glutamylisoleucine*	LC/MS pos early	34456	_	HMDB11170	14253342	100001485	_	2940	261.1445
gamma-glutamylleucine	LC/MS neg	18369	_	HMDB11171	151023	1268	2566-39-4	1685	259.12994
gamma-glutamyl-alpha-lysine	LC/MS pos early	55015	_	_	65254	100010901	_	2784	276.1554
gamma-glutamyl-epsilon-lysine	LC/MS pos early	33934	_	HMDB03869	7015684;7015685	100001262	17105-15-6	2717	276.1554
gamma-glutamylmethionine	LC/MS pos early	44872	_	HMDB29155	7009567	100001313	17663-87-5	2640	279.10092
gamma-glutamylphenylalanine	LC/MS neg	33422	_	HMDB00594	111299	100000491	7432-24-8	1825	293.11429
gamma-glutamylthreonine	LC/MS pos early	33364	_	HMDB29159	_	100001314	5652-48-2	1750	249.10812
gamma-glutamyltyrosine	LC/MS neg	2734	_	HMDB11741	94340	1102	7432-23-7	1240	309.10921
gamma-glutamylvaline	LC/MS pos early	43829	_	HMDB11172	7015683	100001126	2746-34-1	2700	247.12885
gamma-glutamyl-2-aminobutyrate	LC/MS pos early	37092	_	_	_	100001502	16869-42-4	2380	233.1132
carnosine	LC/MS pos early	1768	C00386	HMDB00033	439224	249	305-84-0	2942	227.11387
alanylleucine	LC/MS pos early	37093	_	HMDB28691	259583	100001890	1638-60-4	3055	203.13902
glycylisoleucine	LC/MS neg	36659	_	HMDB28844	88079	100001790	19461-38-2	1820	187.10881
glycylleucine	LC/MS pos early	34398	C02155	HMDB00759	92843	100001258	869-19-2	3059	189.12337
glycylvaline	LC/MS neg	18357	_	HMDB28854	97417	100000487	1963-21-9	1371.1	173.09316
isoleucylglycine	LC/MS neg	40008	_	_	342532	100003169	868-28-0	1992	187.10881
leucylalanine	LC/MS pos early	40010	_	_	259321	100003179	7298-84-2	2801	203.13902
leucylglycine	LC/MS pos early	40045	_	_	79070	100003185	686-50-0	2778	189.12337
phenylalanylalanine	LC/MS pos early	41374	_	_	6993123;5488196	100003589	3918-87-4	2909	237.12337
phenylalanylglycine	LC/MS pos early	41370	_	_	98207	100003588	721-90-4	2898	223.10772
prolylglycine	LC/MS pos early	40703	_	_	7408076;6426709	100003674	2578-57-6	2136	173.09207
threonylphenylalanine	LC/MS neg	31530	_	_	4099799;4099798	100001125	16875-27-7	2322	265.11938
tryptophylglycine	LC/MS neg	43028	_	_	263471	100003609	7360-09-0	2481	260.10406
tyrosylglycine	LC/MS neg	41375	_	_	259323	100003598	673-05-5	1635	237.08808
valylglutamine	LC/MS pos early	42079	_	_	5253209	100003640	42854-54-6	2295	246.14484
valylglycine	LC/MS neg	40475	_	HMDB29127	136487	100003641	686-43-1	1506	173.09316
valylleucine	LC/MS pos early	39994	_	HMDB29131	352039	100003210	22906-55-4	3138	231.17032
leucylglutamine*	LC/MS pos early	48189	_	_	4305457	100003183	_	2620	260.1605
Ac-Ser-Asp-Lys-Pro-OH	LC/MS neg	40707	_	_	4409396	100003761	127103-11-1	1290	486.22055
phenylacetylglycine	LC/MS neg	33945	C05598	HMDB00821	68144	100001275	500-98-1	2375	192.06661
glucose	LC/MS polar	48152	C00031	HMDB00122	79025	572	50-99-7	2342	225.06159
glucose 6-phosphate	LC/MS polar	31260	C00668	HMDB01401	5958	291	103192-55-8	4537	259.02244

Isobar: fructose 1,6-diphosphate, glucose 1,6-diphosphate, myo-inositol 1,4 or 1,3-diphosphate	LC/MS neg	46896	-	-	-	100002180	-	551	338.98897
dihydroxyacetone phosphate (DHAP)	LC/MS pos early	15522	C00111	HMDB01473	668	309	102783-56-2	539	171.00531
2-phosphoglycerate	LC/MS polar	35629	C00631	HMDB03391	59	100000093	70195-25-4	4346	184.98566
3-phosphoglycerate	LC/MS neg	1414	C00597	HMDB00807	724	132	80731-10-8	583	184.98566
phosphoenolpyruvate (PEP)	LC/MS neg	597	C00074	HMDB00263	1005	463	10526-80-4	587.3	166.9751
pyruvate	LC/MS polar	22250	C00022	HMDB00243	1060	823	127-17-3	2650	175.02481
lactate	LC/MS neg	527	C00186	HMDB00190	612	482	79-33-4	681.6	89.02442
glycerate	LC/MS polar	1572	C00258	HMDB00139	752	1052	600-19-1	2070.4	105.01933
6-phosphogluconate	LC/MS neg	15442	C00345	HMDB01316	91493	100000341	921-62-0;53411-70-4	583.9	275.01735
ribose 5-phosphate	LC/MS polar	561	C00117	HMDB01548	-	19	18265-46-8;108321-05-7	4193	229.01188
ribose 1-phosphate	LC/MS polar	1763	C00620	HMDB01489	439236	219	50-99-7;58459-37-3	4025	229.01188
5-phosphoribosyl diphosphate (PRPP)	LC/MS neg	36840	C00119	HMDB00280	7339	166	108321-05-7	570	308.97822
sedoheptulose-7-phosphate	LC/MS neg	35649	C05382	HMDB01068	616	100001628	2646-35-7	599.6	289.033
ribulose/xylulose 5-phosphate	LC/MS polar	37288	C00199	-	-	100002179	-	3888	229.01187
ribose	LC/MS polar	1471	C00121	HMDB00283	5779	914	50-69-1	1508.2	195.05103
ribitol	LC/MS polar	15772	C00474	HMDB00508	6912	100000406	488-81-3	1789.6	151.06119
ribonate	LC/MS polar	27731	C01685	HMDB00867	5460677	100001007	8/3/36	2425	165.04046
arabitol/xylitol	LC/MS polar	48885	-	-	-	100006430	-	1932.4	151.0612
ribulose/xylulose	LC/MS polar	48340	-	-	-	100006122	-	1400	149.0455
arabonate/xylonate	LC/MS polar	48255	-	-	-	100006115	-	2664.6	165.04046
sedoheptulose	LC/MS polar	53237	-	HMDB03219	5459879	100002619	3019-74-7	2255	209.06667
maltopentaose	LC/MS neg	35163	C06218	HMDB12254	13489094	100001447	34620-76-3	1080.9	827.2674
maltotetraose	LC/MS neg	15910	C02052	HMDB01296	446495	100000275	34612-38-9	910	665.21458
maltotriose	LC/MS polar	44688	C01835	HMDB01262	439586	100000276	1109-28-0	4286.4	549.16723
maltose	LC/MS polar	15586	C00208	HMDB00163	10991489	913	6363-53-7	3329.4	387.11442
lactose	LC/MS polar	567	C00243	HMDB00186	84571	393	5965-66-2	3500	387.11442
3-sialyllactose	LC/MS neg	40424	-	HMDB00825	123914	100003408	35890-38-1	730	632.20435
fructose	LC/MS polar	577	C00095	HMDB00660	5984	878	57-48-7	2022.2	179.05611
mannitol/sorbitol	LC/MS polar	46142	C01507	HMDB00247	5780	100001740	-	2260	181.07176
mannose	LC/MS polar	584	C00159	HMDB00169	18950	803	3458-28-4	2200	179.05611
mannose-6-phosphate	LC/MS polar	1469	C00275	HMDB01078	439198	294	70442-25-0;104872-94-8	4510	259.02244
galactitol (dulcitol)	LC/MS polar	1117	C01697	HMDB00107	11850	1003	608-66-2	2309.7	181.07176
galactose 1-phosphate	LC/MS polar	15706	C00446	HMDB00645	123912	100000300	19046-60-7	4552	259.02244
galactonate	LC/MS polar	27719	C00880	HMDB00565	128869	100001026	299-28-5	3085	195.05102
UDP-glucose	LC/MS polar	32344	C00029	HMDB00286	8629	1096	117756-22-6	3950	565.04775
UDP-galactose	LC/MS polar	15860	C00052	HMDB00302	18068	100000298	2956-16-3;91183-98-1;137868-52-1	4000	565.04774
UDP-glucuronate	LC/MS neg	2763	C00167	HMDB00935	17473	1097	28053-08-9;63700-19-6	588	579.02701
guanosine 5'-diphospho-fucose	LC/MS polar	15903	-	-	-	335	15839-70-0	4225	588.07497

UDP-N-acetylglucosamine	LC/MS polar	35162	C00043	HMDB00290	445675	1249	91183-98-1	3708.7	606.0743
UDP-N-acetylgalactosamine	LC/MS polar	18396	C00203	HMDB00304	439185	1257	108320-87-2	3730	606.0743
cytidine 5'-monophospho-N-acetylneuraminic acid	LC/MS polar	36831	C00128	HMDB01176	448209	1223	3063-71-6	3900	613.13999
glucuronate	LC/MS polar	15443	C00191	HMDB00127	444791	100000257	207300-70-7	3233.3	193.03537
N-acetylglucosamine 6-phosphate	LC/MS polar	15107	C00357	HMDB02817	439219	1213	102029-88-9	4114	300.04899
N-acetyl-glucosamine 1-phosphate	LC/MS polar	15741	C04256	HMDB01367	440364	1212	31281-59-1	4052	300.04899
N-acetylneuraminate	LC/MS polar	32377	C00270	HMDB00230	439197	1162	131-48-6	3072.6	308.0987
N-acetylglucosaminylasparagine	LC/MS pos early	48149	C04540	HMDB00489	123826	1215	2776-93-4	1212	336.14015
erythronate*	LC/MS polar	42420	-	HMDB00613	2781043	100001320	88759-55-1	2186	135.02989
N-acetylglucosamine/N-acetylgalactosamine	LC/MS pos early	46539	-	-	-	100006435	-	715	222.0972
citrate	LC/MS neg	1564	C00158	HMDB00094	311	1124	77-92-9	582	191.01973
aconitate [cis or trans]	LC/MS neg	46173	C00417	HMDB00072	-	100001359	-	580	173.00916
isocitrate	LC/MS polar	12110	C00311	HMDB00193	1198	1206	20226-99-7	4400	191.01973
alpha-ketoglutarate	LC/MS polar	528	C00026	HMDB00208	51	93	305-72-6;328-50-7;22202-68-2	2700	145.01425
succinyl CoA	LC/MS neg	36857	C00091	HMDB01022	439161	511	108347-97-3	1450	432.55835
succinylcarnitine (C4-DC)	LC/MS pos early	37058	-	-	-	100001948	256928-74-2	2291	262.12852
succinate	LC/MS polar	1437	C00042	HMDB00254	1110	252	110-15-6	3149	117.01933
fumarate	LC/MS polar	1643	C00122	HMDB00134	444972	330	100-17-8	3084	115.00368
malate	LC/MS polar	1303	C00149	HMDB00156	525	409	6915-15-7	3343.1	133.01425
2-methylcitrate/homocitrate	LC/MS neg	52282	-	-	-	100008929	-	575	205.03538
acetylphosphate	LC/MS polar	15488	C00227	HMDB01494	186	1211	94249-01-1	4013.9	138.98018
phosphate	LC/MS neg	42109	C00009	HMDB01429	1061	461	7664-38-2	608	96.96962
laurate (12:0)	LC/MS neg	1645	C02679	HMDB00638	3893	181	143-07-7	5300	199.17035
5-dodecenoate (12:1n7)	LC/MS neg	33968	-	HMDB00529	5312378	100001232	2430-94-6	5224	197.1547
myristate (14:0)	LC/MS neg	1365	C06424	HMDB00806	11005	519	544-63-8	5440	227.20165
myristoleate (14:1n5)	LC/MS neg	32418	C08322	HMDB02000	5281119	100001198	544-64-9	5346.9	225.186
pentadecanoate (15:0)	LC/MS neg	1361	C16537	HMDB00826	13849	980	1002-84-2;10002-84-2	5521	241.2173
palmitate (16:0)	LC/MS neg	1336	C00249	HMDB00220	985	424	57-10-3	5618	255.23295
palmitoleate (16:1n7)	LC/MS neg	33447	C08362	HMDB03229	445638	452	373-49-9	5475	253.2173
margarate (17:0)	LC/MS neg	1121	-	HMDB02259	10465	891	506-12-7	5731	269.2486
10-heptadecenoate (17:1n7)	LC/MS neg	33971	-	HMDB60038	5312435	100001278	29743-97-3	5555	267.23295
stearate (18:0)	LC/MS neg	1358	C01530	HMDB00827	5281	439	57-11-4	5872	283.26425
nonadecanoate (19:0)	LC/MS neg	1356	C16535	HMDB00772	12591	892	646-30-0	6068	297.2799
10-nonadecenoate (19:1n9)	LC/MS neg	33972	-	HMDB13622	5312513	100001277	73033-09-7	5780	295.26425
arachidate (20:0)	LC/MS neg	1118	C06425	HMDB02212	10467	893	506-30-9	6295	311.29555
eicosenoate (20:1)	LC/MS neg	33587	-	HMDB02231	5282768	100001335	-	5950	309.2799
erucate (22:1n9)	LC/MS neg	1552	C08316	HMDB02068	5281116	1087	112-86-7	6355.6	337.3112
oleate/vaccenate (18:1)	LC/MS neg	52285	-	-	-	100008930	-	5655	281.2486
stearidonate (18:4n3)	LC/MS neg	33969	C16300	HMDB06547	5312508	100001229	111174-40-4	5395	275.20165
eicosapentaenoate (EPA; 20:5n3)	LC/MS neg	18467	C06428	HMDB01999	446284	2050	10-2005-9;10417-94-4	5450	301.2173

docosapentaenoate (n3 DPA; 22:5n3)	LC/MS neg	32504	C16513	HMDB01976	6441454	100001181	2234-74-4	5571	329.2486
docosahexaenoate (DHA; 22:6n3)	LC/MS neg	44675	C06429	HMDB02183	445580	100000665	6217-54-5	5525	327.23295
docosatrienoate (22:3n3)	LC/MS neg	32417	C16534	HMDB02823	5312556	100001195	59708-86-0	5823	333.2799
linoleate (18:2n6)	LC/MS neg	1105	C01595	HMDB00673	5280450	180	60-33-3	5535	279.23295
linolenate [alpha or gamma; (18:3n3 or 6)]	LC/MS neg	34035	C06426	HMDB03073	5280934	100001337	_	5450	277.2173
dihomo-linolenate (20:3n3 or n6)	LC/MS neg	35718	C03242	HMDB02925	5280581	100001739	17046-59-2	5596	305.2486
arachidonate (20:4n6)	LC/MS neg	1110	C00219	HMDB01043	444899	229	506-32-1	5535	303.23295
adrenate (22:4n6)	LC/MS neg	32980	C16527	HMDB02226	5497181	100001193	2091-25-0	5678	331.26425
docosapentaenoate (n6 DPA; 22:5n6)	LC/MS neg	37478	C16513	HMDB01976	6441454	100001580	25182-74-5	5624.5	329.2486
docosadienoate (22:2n6)	LC/MS neg	32415	C16533	HMDB61714	5282807	100001182	7370-49-2	6034	335.29555
dihomo-linoleate (20:2n6)	LC/MS neg	17805	C16525	HMDB05060	6439848	1231	2091-39-6	5730	307.26425
mead acid (20:3n9)	LC/MS neg	35174	_	HMDB10378	5312531	100001472	20590-32-3	5650	305.2486
docosatrienoate (22:3n6)*	LC/MS neg	57467	_	_	_	100015762	_	5850	333.27989
15-methylpalmitate	LC/MS neg	38768	_	_	17903417	100002945	_	5695	269.24868
17-methylstearate	LC/MS neg	38296	_	_	3083779	100002356	2724-59-6	5993	297.2799
dimethylmalonic acid	LC/MS polar	42978	_	HMDB02001	11686	100004251	595-46-0	2702	131.03498
2-hydroxyglutarate	LC/MS polar	37253	C02630	HMDB00606	43	100002070	40951-21-1	3352.8	147.02989
adipate	LC/MS polar	21134	C06104	HMDB00448	196	100000863	124-04-9	3000	145.05063
2-hydroxyadipate	LC/MS polar	31934	C02360	HMDB00321	193530	100001153	18294-85-4	3000	161.04554
maleate	LC/MS polar	20676	C01384	HMDB00176	444266	100000707	110-16-7	2510	115.00368
hexadecanedioate	LC/MS neg	35678	C19615	HMDB00672	10459	100001614	505-54-4	4615	285.20713
1-dihomo-linoleoylglycerol (20:2)	LC/MS neg	35103	_	_	_	100001483	_	6800	307.26423
malonylcarnitine	LC/MS pos early	37059	_	HMDB02095	22833583	100001526	853728-01-5	2086	248.11287
malonate	LC/MS polar	15872	C00383	HMDB00691	867	818	141-82-2;26522-22-85-0	3447	103.00368
acetyl CoA	LC/MS neg	43840	C00024	HMDB01206	444493	1830	102029-73-2	1691	403.55561
butyrylcarnitine (C4)	LC/MS pos early	32412	C02862	HMDB02013	439829	100001054	25576-40-3	2860	232.15434
propionyl CoA	LC/MS neg	46323	C00100	HMDB01275	92753	100000355	108321-21-7	2009	410.56343
propionylcarnitine (C3)	LC/MS pos early	32452	C03017	HMDB00824	107738	100001162	17298-37-2	2590	218.13869
methylmalonate (MMA)	LC/MS polar	1496	C02170	HMDB00202	487	418	516-05-2	3078.4	117.01933
N-palmitoylglycine	LC/MS neg	42092	_	_	151008	100003686	2441-41-0	5580	312.25441
acetylcarnitine (C2)	LC/MS pos early	32198	C02571	HMDB00201	1	100000802	5080-50-2	2282	204.12304
3-hydroxybutyrylcarnitine (1)	LC/MS pos early	43264	_	HMDB13127	53481617	100003926	_	2400	248.14925
3-hydroxybutyrylcarnitine (2)	LC/MS pos early	52984	_	_	_	100009271	_	2340	248.14925
valerylcarnitine (C5)	LC/MS pos early	34406	_	HMDB13128	6426903	100001394	40225-14-7	3112	246.16999
hexanoylcarnitine (C6)	LC/MS pos early	32328	_	HMDB00705	6426853	100000781	6920-35-0	3308	260.18564
octanoylcarnitine (C8)	LC/MS pos late	33936	C02838	HMDB00791	123701	100001247	3671-77-0	950	288.21694
decanoylcarnitine (C10)	LC/MS pos late	33941	_	HMDB00651	10245190	100001251	1492-27-9	1130	316.24824
cis-4-decenoylcarnitine (C10:1)	LC/MS pos late	38178	_	_	_	100002259	98930-66-6	1057	314.23259
laurylcarnitine (C12)	LC/MS pos late	34534	_	HMDB02250	10427569	100001392	25518-54-1	1235	344.27954
myristoylcarnitine (C14)	LC/MS pos late	33952	_	HMDB05066	53477791	100001270	18822-89-4	1350	372.31084
palmitoylcarnitine (C16)	LC/MS pos late	44681	C02990	HMDB00222	461	100000776	6865-14-1	1425	400.34214

palmitoleoylcarnitine (C16:1)*	LC/MS pos late	53223	_	_	_	100009406	_	1357	398.32649
stearoylcarnitine (C18)	LC/MS pos late	34409	_	HMDB00848	6426855	100001391	18822-91-8	1485	428.37344
linoleoylcarnitine (C18:2)*	LC/MS pos late	46223	_	HMDB06469	6450015	100003151	36816-10-1	1430	424.34214
linolenoylcarnitine (C18:3)*	LC/MS pos late	57511	_	_	_	100015831	_	1310	422.32649
oleoylcarnitine (C18:1)	LC/MS pos late	35160	_	HMDB05065	6441392;53477789	100001501	38677-66-6	1423	426.35779
myristoleoylcarnitine (C14:1)*	LC/MS pos late	48182	_	_	_	100006051	889848-55-9	1316	370.29471
adipoylcarnitine (C6-DC)	LC/MS pos early	52988	_	_	_	100006614	_	2558	290.15982
arachidoylcarnitine (C20)*	LC/MS pos late	57513	_	HMDB06460	_	100015833	_	1512	456.40474
arachidonoylcarnitine (C20:4)	LC/MS pos late	57518	_	_	_	100015837	_	1353	448.34215
adrenoylcarnitine (C22:4)*	LC/MS pos late	57528	_	_	_	100015850	_	1415	476.37343
behenoylcarnitine (C22)*	LC/MS pos late	57514	_	_	_	100015832	_	1561	484.43604
dihomo-linolenoylcarnitine (20:3n3 or 6)*	LC/MS pos late	57521	_	_	_	100015840	_	1392	450.35777
dihomo-linoleoylcarnitine (C20:2)*	LC/MS pos late	57520	_	_	_	100015839	_	1436	452.37343
eicosenoylcarnitine (C20:1)*	LC/MS pos late	57519	_	_	_	100015838	_	1484	454.38909
erucoylcarnitine (C22:1)*	LC/MS pos late	57525	_	_	_	100015841	_	1535	482.4204
docosadienoylcarnitine (C22:2)*	LC/MS pos late	57526	_	_	_	100015848	_	1493	480.40474
docosatrienoylcarnitine (C22:3)*	LC/MS pos late	57527	_	_	_	100015849	_	1460	478.38909
docosapentaenoylcarnitine (C22:5n3)*	LC/MS pos late	57529	_	_	_	100015851	_	1387	474.35777
docosahexaenoylcarnitine (C22:6)*	LC/MS pos late	57523	_	_	_	100015845	_	1349	472.34215
tetracosadienoylcarnitine (C24:2)*	LC/MS pos late	57524	_	_	_	100015847	_	1542	508.43605
lignoceroylcarnitine (C24)*	LC/MS pos late	57515	_	_	_	100015834	_	1603	512.46733
margaroylcarnitine*	LC/MS pos late	57512	_	_	_	100004054	106182-29-0	1424	414.35779
nervonoylcarnitine (C24:1)*	LC/MS pos late	57531	_	_	_	100015846	_	1602	510.45168
cerotoylcarnitine (C26)*	LC/MS pos late	57516	_	HMDB06347	_	100015835	_	1649	540.49864
ximenoylcarnitine (C26:1)*	LC/MS pos late	57517	_	_	_	100015836	_	1607	538.48299
deoxycarnitine	LC/MS pos early	36747	C01181	HMDB01161	134	100001662	6249-56-5	2052	146.11756
carnitine	LC/MS pos early	15500	C00318	HMDB00062	10917	100000007	461-05-2	1978	162.11247
3-hydroxybutyrate (BHBA)	LC/MS polar	542	C01089	HMDB00357	441	254	625-72-9	1443.3	103.04007
acetylcholine	LC/MS pos early	18790	_	_	_	1749	60-32-1	2220	146.11756
2-hydroxydecanoate	LC/MS neg	42489	_	_	21488	100004089	5393-81-7	4840	187.13396
2-hydroxypalmitate	LC/MS neg	35675	_	HMDB31057	92836	100001579	764-67-0	5511.2	271.22786
2-hydroxystearate	LC/MS neg	17945	C03045	_	69417	1239	629-22-1	5695	299.25917
3-hydroxyhexanoate	LC/MS neg	53230	_	_	151492	100006367	10191-24-9	1725	131.07136
3-hydroxyoctanoate	LC/MS neg	22001	_	HMDB01954	26613	100000773	88930-08-9	3446	159.10266
3-hydroxydecanoate	LC/MS neg	22053	_	HMDB02203	26612	100000997	5561-87-5	4634.7	187.13396
3-hydroxylaurate	LC/MS neg	32457	_	HMDB00387	94216	100001112	53941-38-1	5175	215.16526
3-hydroxypalmitate	LC/MS neg	27503	_	HMDB10734	301590	100000983	928-17-6	5476	271.22786
16-hydroxypalmitate	LC/MS neg	39609	C18218	HMDB06294	10466	100002953	506-13-8	5236.2	271.22786
13-HODE + 9-HODE	LC/MS neg	37752	_	_	43013	100002196	_	5275	295.22825
prostaglandin E2	LC/MS neg	7746	C00584	HMDB01220	5280360	487	363-24-6	4573	351.2177
prostaglandin F2alpha	LC/MS neg	19398	C00639	HMDB01139	5280363	100000574	551-11-1	4658	353.23334

oleoyl ethanolamide	LC/MS neg	38102	_	HMDB02088	5283454	1137	11-58-0;111-58-0	6450	324.2908
palmitoyl ethanolamide	LC/MS neg	38165	C16512	HMDB02100	4671	1489	544-31-0	6300	298.27515
stearoyl ethanolamide	LC/MS pos late	38625	_	HMDB13078	27902	100002254	111-57-9	1787	328.32101
N-oleoyltaurine	LC/MS neg	39732	_	_	6437033	100003119	52514-04-2	5610	388.2527
N-stearoyltaurine	LC/MS neg	39730	_	_	168274	100003240	63155-80-6	5782	390.26835
N-palmitoyltaurine	LC/MS neg	39835	_	_	_	100003239	83982-06-3	5560	362.23705
linoleoyl ethanolamide	LC/MS neg	52608	_	HMDB12252	5283446	100006726	68171-52-8	6150	322.27515
lignoceroyl ethanolamide (24:0)*	LC/MS pos late	57535	_	_	_	100015855	_	2445	412.41491
palmitoleoyl ethanolamide*	LC/MS pos late	57541	_	HMDB13648	9835868	100015862	_	1546	298.27406
ximenoyl ethanolamide (26:1)*	LC/MS pos late	57537	_	_	_	100015859	_	2425	438.43056
myo-inositol	LC/MS polar	1124	C00137	HMDB00211	892	363	87-89-8	3506.3	225.06159
inositol 1-phosphate (I1P)	LC/MS polar	43849	C04006	HMDB00213	440194	370	573-35-3;106032-59-1	4785	259.02244
choline	LC/MS pos early	15506	C00114	HMDB00097	305	1256	67-48-1	1961	104.10699
choline phosphate	LC/MS pos early	34396	C00588	HMDB01565	1014	267	72556-74-2	700	184.07332
cytidine 5'-diphosphocholine	LC/MS pos early	34418	C00307	HMDB01413	13804	1220	33818-15-4	725	489.11461
glycerophosphorylcholine (GPC)	LC/MS pos early	15990	C00670	HMDB00086	71920	100000269	28319-77-9	672	258.1101
phosphoethanolamine	LC/MS polar	1600	C00346	HMDB00224	1015	1026	1071-23-4	4559.1	140.01182
cytidine-5'-diphosphoethanolamine	LC/MS polar	34410	C00570	HMDB01564	123727	100001407	72842-05-8	4210	445.0531
glycerophosphoethanolamine	LC/MS polar	37455	C01233	HMDB00114	123874	100001620	33049-08-0	3075	214.04859
glycerophosphoserine*	LC/MS pos early	57404	_	_	3081457	100015666	_	577	260.05298
trimethylamine N-oxide	LC/MS pos early	40406	C01104	HMDB00925	1145	100003397	1184-78-7	2100	76.07569
glycerophosphoinositol*	LC/MS polar	47155	_	_	_	100001621	16824-65-0	3850	333.05922
1,2-dipalmitoyl-GPC (16:0/16:0)	LC/MS pos late	19130	_	HMDB00564	452110	100000657	63-89-8	2365	734.56944
1,2-dipalmitoyl-GPE (16:0/16:0)*	LC/MS pos late	57341	_	_	11802774;445468	100009204	_	2498	692.52248
1-palmitoyl-2-oleoyl-GPA (16:0/18:1)	LC/MS pos late	19262	C13889	_	5283523	1535	169437-35-8	3293	692.52249
1-palmitoyl-2-oleoyl-GPC (16:0/18:1)	LC/MS pos late	52461	_	_	6436017	1539	26853-31-6;26853-31-6	2358	760.58509
1-palmitoyl-2-linoleoyl-GPC (16:0/18:2)	LC/MS pos late	42446	_	_	5287971	1537	40811-94-7	2160	758.56944
1-stearoyl-2-arachidonoyl-GPC (18:0/20:4)	LC/MS pos late	42450	_	_	16219824	100001869	35418-59-8	2300	810.60074
1-stearoyl-2-oleoyl-GPC (18:0/18:1)	LC/MS pos late	52438	_	_	_	100008904	56421-10-4	2644	788.61639
1-stearoyl-2-oleoyl-GPI (18:0/18:1)*	LC/MS pos late	52726	_	_	_	100009181	_	3711	882.60661
1,2-dioleoyl-GPC (18:1/18:1)	LC/MS pos late	52457	_	_	10350317	100008905	4235-95-4	2346	786.60074
1-palmitoyl-2-arachidonoyl-GPC (16:0/20:4n6)	LC/MS pos late	52462	_	_	10747814	100008914	35418-58-7	2091	782.56944
1-stearoyl-2-linoleoyl-GPC (18:0/18:2)*	LC/MS pos late	52452	_	_	_	100008980	_	2380	786.60074
1-palmitoleoyl-2-oleoyl-GPC (16:1/18:1)*	LC/MS pos late	52458	_	_	_	100008983	_	2155	758.56944
1-palmitoyl-2-palmitoleoyl-GPC (16:0/16:1)*	LC/MS pos late	52470	_	_	_	100008984	_	2160	732.55378
1,2-dipalmitoleoyl-GPC (16:1/16:1)*	LC/MS pos late	52472	_	_	_	100008997	_	1992	730.53813
1-stearoyl-2-arachidonoyl-GPI (18:0/20:4)	LC/MS pos late	52449	_	_	_	100000616	383907-33-3	3000	904.59096
1-oleoyl-2-linoleoyl-GPC (18:1/18:2)*	LC/MS pos late	52453	_	_	_	100008981	_	2165	784.58509
1-palmitoyl-2-arachidonoyl-GPI (16:0/20:4)*	LC/MS pos late	52467	_	_	_	100008993	_	2567	876.55966
1-palmitoleoyl-2-oleoyl-GPI (16:1/18:1)*	LC/MS pos late	52725	_	_	_	100009174	_	2665	852.55966
1-palmitoyl-2-oleoyl-GPG (16:0/18:1)	LC/MS pos late	52448	_	_	5283509	100000641	_	3206	766.55927

1-palmitoleoyl-2-oleoyl-GPG (16:1/18:1)*	LC/MS pos late	57398	_	_	_	100009106	_	2737	764.54362
1-palmitoleoyl-2-oleoyl-GPE (16:1/18:1)*	LC/MS pos late	52469	_	_	_	100008995	_	2250	716.52248
1-palmitoyl-2-oleoyl-GPE (16:0/18:1)	LC/MS pos late	19263	_	HMDB05320	5283496	1526	26662-94-2	2509	718.53813
1-stearoyl-2-arachidonoyl-GPE (18:0/20:4)	LC/MS pos late	52447	_	_	5289133	100008977	_	2424	768.55378
1-stearoyl-2-oleoyl-GPE (18:0/18:1)	LC/MS pos late	42448	_	_	_	100001856	_	2858	746.56944
1-palmitoyl-2-arachidonoyl-GPE (16:0/20:4)*	LC/MS pos late	52464	_	HMDB05323	9546800	100008990	_	2198	740.52248
1-palmitoyl-2-linoleoyl-GPE (16:0/18:2)	LC/MS pos late	42449	_	HMDB05322	9546747	100001870	_	2275	716.52248
1-stearoyl-2-linoleoyl-GPE (18:0/18:2)*	LC/MS pos late	52446	_	_	9546749	100008976	_	2522	744.55378
1,2-dioleoyl-GPG (18:1/18:1)	LC/MS polar	19142	_	_	11846228	1541	67254-28-8	715	773.53381
1,2-dioleoyl-GPI (18:1/18:1)	LC/MS polar	52619	_	_	_	100008908	_	913	861.54985
1-palmitoyl-2-stearoyl-GPC (16:0/18:0)	LC/MS pos late	52616	_	_	_	100008921	59403-51-9	2653	762.60074
1,2-dioleoyl-GPE (18:1/18:1)	LC/MS pos late	52609	_	_	9546757	100008906	5/1/04	2485	744.55378
1-palmitoyl-2-oleoyl-GPI (16:0/18:1)*	LC/MS pos late	52669	_	_	_	100009066	_	3140	854.57531
1,2-dioleoyl-GPS (18:1/18:1)	LC/MS pos late	19191	_	_	6438639	1533	6811-55-8	2800	788.54361
1-stearoyl-2-oleoyl-GPG (18:0/18:1)	LC/MS pos late	52623	_	_	_	100001866	322647-48-3	3714	794.59057
1,2-dilinoleoyl-GPC (18:2/18:2)	LC/MS pos late	52603	_	_	5288075	100008903	998-06-1	2006	782.56944
1-linoleoyl-2-arachidonoyl-GPC (18:2/20:4n6)*	LC/MS pos late	52710	_	_	_	100009131	_	1969	806.56944
1-palmitoyl-2-oleoyl-GPS (16:0/18:1)	LC/MS pos late	19261	C13880	_	5283499	1531	40290-44-6	2660	762.52797
1,2-dipalmitoyl-GPG (16:0/16:0)	LC/MS pos late	52625	_	_	11846227	100000652	200880-41-7	3157	740.54362
1-oleoyl-2-arachidonoyl-GPE (18:1/20:4)*	LC/MS pos late	55041	_	_	_	100009219	_	2200	766.53813
1-oleoyl-2-arachidonoyl-GPI (18:1/20:4) *	LC/MS pos late	54994	_	_	_	100010951	_	2568	902.57531
1,2-dipalmitoleoyl-GPE (16:1/16:1)*	LC/MS pos late	52688	_	HMDB05342	9546809	1525	_	2063	688.49119
1-palmitoyl-2-gamma-linolenoyl-GPC (16:0/18:3n6)*	LC/MS pos late	54812	_	_	_	100009154	_	2034	756.55378
1-stearoyl-2-arachidonoyl-GPS (18:0/20:4)	LC/MS pos late	52235	_	_	_	100001872	_	2565	812.54361
1-stearoyl-2-oleoyl-GPS (18:0/18:1)	LC/MS pos late	19265	_	_	9547087	100000639	_	3000	790.55926
1-palmitoyl-GPC (16:0)	LC/MS pos late	33955	_	HMDB10382	86554	100001263	17364-16-8	1525	496.33977
2-palmitoyl-GPC (16:0)*	LC/MS pos late	35253	_	HMDB61702	15061532	100001562	_	1505	496.33977
1-palmitoleoyl-GPC (16:1)*	LC/MS pos late	33230	_	HMDB10383	24779461	100001511	_	1450	494.32412
2-palmitoleoyl-GPC (16:1)*	LC/MS pos late	35819	_	_	_	100001561	_	1425	494.32412
1-stearoyl-GPC (18:0)	LC/MS pos late	33961	_	HMDB10384	497299	100001271	19420-57-6	1606	524.37107
1-oleoyl-GPC (18:1)	LC/MS pos late	48258	_	HMDB02815	16081932	100001272	19420-56-5	1540	522.35542
1-linoleoyl-GPC (18:2)	LC/MS pos late	34419	C04100	HMDB10386	11988421	100001395	_	1465	520.33977
1-arachidonoyl-GPC (20:4n6)*	LC/MS pos late	33228	C05208	HMDB10395	_	100001551	_	1460	544.33977
1-lignoceroyl-GPC (24:0)	LC/MS pos late	49617	_	_	_	100002873	325171-59-3	1907	608.46497
1-palmitoyl-GPE (16:0)	LC/MS pos late	35631	_	HMDB11503	9547069	100001567	53862-35-4	1544	454.29282
1-stearoyl-GPE (18:0)	LC/MS pos late	42398	_	HMDB11130	9547068	100001461	69747-55-3	1626	482.32412
2-stearoyl-GPE (18:0)*	LC/MS neg	41220	_	_	_	100003901	_	6350	480.30956
1-oleoyl-GPE (18:1)	LC/MS pos late	35628	_	HMDB11506	9547071	100001569	89576-29-4	1554	480.30847
1-linoleoyl-GPE (18:2)*	LC/MS pos late	36600	_	HMDB11507	52925130	100001570	_	1482	478.29282
1-arachidonoyl-GPE (20:4n6)*	LC/MS neg	35186	_	HMDB11517	42607465	100001571	_	5874	500.27826
1-palmitoyl-GPI (16:0)	LC/MS neg	35305	_	HMDB61695	_	100001655	_	5564	571.28888

1-stearoyl-GPI (18:0)	LC/MS neg	19324	_	HMDB61696	_	100000656	796963-93-4	5794.3	599.32018
1-oleoyl-GPI (18:1)*	LC/MS neg	36602	_	_	_	100001777	_	5599	597.30453
1-linoleoyl-GPI (18:2)*	LC/MS neg	36594	_	_	_	100001778	_	5494	595.28888
1-arachidonoyl-GPI (20:4)*	LC/MS neg	34214	_	HMDB61690	_	100001654	_	5482	619.28888
1-stearoyl-GPS (18:0)*	LC/MS neg	45966	_	_	9547101	100004327	_	5800	524.29939
1-oleoyl-GPS (18:1)	LC/MS neg	19260	_	_	9547099	100000630	326589-90-6	5607	522.28374
1-palmitoyl-GPA (16:0)	LC/MS pos late	34428	C04036	HMDB00327	6419701	100001445	17618-08-5	1625	393.24006
1-stearoyl-GPA (18:0)	LC/MS pos late	36810	_	_	89567	100001670	325465-92-7;799268-65-8	1739	439.28192
1-palmitoyl-GPG (16:0)*	LC/MS neg	45970	_	_	3300276	100005717	_	5570	483.27284
1-palmitoyl-GPS (16:0)*	LC/MS neg	46130	_	_	9547100	100005805	_	5572	496.26809
1-stearoyl-GPG (18:0)	LC/MS neg	34437	_	_	_	100001462	_	5816	511.30414
1-oleoyl-GPG (18:1)*	LC/MS pos late	45968	_	_	_	100005716	_	1628	511.30292
1-linoleoyl-GPG (18:2)*	LC/MS neg	54885	_	_	_	100009227	_	5516	507.27284
1-(1-enyl-palmitoyl)-2-oleoyl-GPE (P-16:0/18:1)*	LC/MS pos late	52477	_	_	_	100009005	_	2600	702.54322
1-(1-enyl-palmitoyl)-2-linoleoyl-GPE (P-16:0/18:2)*	LC/MS pos late	52677	_	_	_	100009069	_	2351	700.52757
1-(1-enyl-palmitoyl)-2-palmitoyl-GPC (P-16:0/16:0)*	LC/MS pos late	52716	_	_	11146967	100009162	_	2454	718.57452
1-(1-enyl-palmitoyl)-2-palmitoleoyl-GPC (P-16:0/16:1)*	LC/MS pos late	52713	_	_	_	100009160	_	2218	716.55887
1-(1-enyl-palmitoyl)-2-arachidonoyl-GPE (P-16:0/20:4)*	LC/MS pos late	52673	_	_	_	100009002	_	2270	724.52757
1-(1-enyl-palmitoyl)-2-oleoyl-GPC (P-16:0/18:1)*	LC/MS pos late	52478	_	_	_	100009007	_	2443	744.59017
1-(1-enyl-stearoyl)-2-oleoyl-GPE (P-18:0/18:1)	LC/MS pos late	52614	_	_	_	100008919	144371-68-6	2950	730.57452
1-(1-enyl-stearoyl)-2-linoleoyl-GPE (P-18:0/18:2)*	LC/MS pos late	52748	_	_	_	100009225	_	2633	728.55887
1-(1-enyl-palmitoyl)-2-arachidonoyl-GPC (P-16:0/20:4)*	LC/MS pos late	52689	_	_	_	100009014	_	2154	766.57452
1-(1-enyl-palmitoyl)-2-linoleoyl-GPC (P-16:0/18:2)*	LC/MS pos late	52682	_	_	_	100009009	_	2226	742.57452
1-(1-enyl-stearoyl)-2-arachidonoyl-GPE (P-18:0/20:4)*	LC/MS pos late	52475	_	HMDB05779	9547058	100008999	_	2511	752.55887
1-(1-enyl-palmitoyl)-GPC (P-16:0)*	LC/MS pos late	52474	_	_	10917802	100002875	_	1547	480.34485
1-(1-enyl-palmitoyl)-GPE (P-16:0)*	LC/MS pos late	39270	_	_	_	100003000	_	1558	438.2979
1-(1-enyl-oleoyl)-GPE (P-18:1)*	LC/MS pos late	44621	_	_	_	100005372	_	1566	464.3136
1-(1-enyl-stearoyl)-GPE (P-18:0)*	LC/MS pos late	39271	_	_	_	100003001	_	1649	466.3292
glycerol	LC/MS pos early	15122	C00116	HMDB00131	753	1254	56-81-5	717	93.05463
glycerol 3-phosphate	LC/MS polar	43847	C00093	HMDB00126	754	100000258	29849-82-9	3938.7	171.00639
glycerophosphoglycerol	LC/MS polar	48857	C03274	_	439964	100001619	_	2430	245.04317
galactosylglycerol*	LC/MS polar	57345	_	_	16048618	100003064	16232-91-0	2518	299.09837
1-myristoylglycerol (14:0)	LC/MS neg	35625	C01885	HMDB11561	79050	100001618	75685-84-6	6353.3	227.20163
2-myristoylglycerol (14:0)	LC/MS neg	34383	_	_	137938	100001408	27214-38-6	6230	227.20163
1-pentadecanoylglycerol (15:0)	LC/MS neg	47898	_	_	190750	100001431	104140-07-0	6200	241.21728
1-palmitoylglycerol (16:0)	LC/MS neg	21127	_	HMDB31074	14900	100000827	542-44-9	6550	255.23293
2-palmitoylglycerol (16:0)	LC/MS neg	33419	_	HMDB11533	123409	100001048	23470-00-0	6400	255.23293
1-oleoylglycerol (18:1)	LC/MS neg	21184	_	HMDB11567	5283468	100000924	111-03-5	6650	281.24858
2-oleoylglycerol (18:1)	LC/MS neg	21232	_	_	5319879	100000943	3443-84-3	6500	281.24858
1-linoleoylglycerol (18:2)	LC/MS neg	27447	_	_	5283469	100001040	2277-28-3	6477	279.23293

2-linoleoylglycerol (18:2)	LC/MS neg	32506	-	HMDB11538	5365676	100000987	3443-82-1	6250	279.23293
1-arachidonylglycerol (20:4)	LC/MS neg	34397	C13857	HMDB11572	5282281	100001433	35474-99-8	6250	303.23293
2-arachidonoylglycerol (20:4)	LC/MS neg	19266	C13856	HMDB04666	5282280	100000584	53847-30-6	6170	303.23293
1-docosahexaenoylglycerol (22:6)	LC/MS neg	35153	-	HMDB11587	-	100001481	-	6150	309.22236
1-dihomo-linolenylglycerol (20:3)	LC/MS neg	48341	-	-	-	100006121	-	6400	305.24858
1-palmitoleoylglycerol (16:1)*	LC/MS neg	52431	-	-	-	100008952	-	6094	253.21727
2-palmitoleoylglycerol (16:1)*	LC/MS neg	52432	-	-	-	100008953	-	6016	253.21727
diacylglycerol (12:0/18:1, 14:0/16:1, 16:0/14:1) [1]*	LC/MS pos late	55002	-	-	-	100010958	-	2636	556.49356
diacylglycerol (12:0/18:1, 14:0/16:1, 16:0/14:1) [2]*	LC/MS pos late	55001	-	-	-	100010959	-	2716	556.49356
diacylglycerol (14:0/18:1, 16:0/16:1) [1]*	LC/MS pos late	54953	-	-	-	100010934	-	3057	584.52486
diacylglycerol (14:0/18:1, 16:0/16:1) [2]*	LC/MS pos late	54954	-	-	-	100010935	-	3183	584.52486
diacylglycerol (16:1/18:2 [2], 16:0/18:3 [1])*	LC/MS pos late	54966	-	-	-	100010940	-	2733	608.52486
oleoyl-linoleoyl-glycerol (18:1/18:2) [1]	LC/MS pos late	46798	-	-	-	100002989	106292-55-1	3115	636.55616
oleoyl-linoleoyl-glycerol (18:1/18:2) [2]	LC/MS pos late	46799	-	-	-	100002990	104346-53-4	3223	636.55616
oleoyl-arachidonoyl-glycerol (18:1/20:4) [1]*	LC/MS pos late	54960	-	-	-	100010936	-	2907	660.55616
oleoyl-arachidonoyl-glycerol (18:1/20:4) [2]*	LC/MS pos late	54961	-	-	-	100010937	-	2990	660.55616
linoleoyl-arachidonoyl-glycerol (18:2/20:4) [1]*	LC/MS pos late	54955	-	-	-	100010922	-	2566	658.54051
linoleoyl-arachidonoyl-glycerol (18:2/20:4) [2]*	LC/MS pos late	54956	-	-	-	100010923	-	2624	658.54051
palmitoyl-dihomo-linolenoyl-glycerol (16:0/20:3n3 or 6) [2]*	LC/MS pos late	54941	-	-	-	100010915	-	3265	636.55616
palmitoyl-arachidonoyl-glycerol (16:0/20:4) [1]*	LC/MS pos late	54957	-	-	-	100010924	-	2916	634.54051
palmitoyl-arachidonoyl-glycerol (16:0/20:4) [2]*	LC/MS pos late	54958	-	-	-	100010925	-	3015	634.54051
palmitoleoyl-linoleoyl-glycerol (16:1/18:2) [1]*	LC/MS pos late	54967	-	-	-	100010930	-	2664	608.52486
palmitoyl-docosahexaenoyl-glycerol (16:0/22:6) [1]*	LC/MS pos late	57373	-	-	-	100015618	-	2749	658.54051
palmitoyl-docosahexaenoyl-glycerol (16:0/22:6) [2]*	LC/MS pos late	57374	-	-	-	100015619	-	2830	658.54051
stearoyl-docosahexaenoyl-glycerol (18:0/22:6) [1]*	LC/MS pos late	57387	-	-	-	100015616	-	3156	686.57181
stearoyl-docosahexaenoyl-glycerol (18:0/22:6) [2]*	LC/MS pos late	57368	-	-	-	100015617	-	3256	686.57181
palmitoyl-myristoyl-glycerol (16:0/14:0) [2]	LC/MS pos late	57364	-	-	-	100015610	-	3180	558.50921
palmitoyl-oleoyl-glycerol (16:0/18:1) [1]*	LC/MS pos late	54943	-	-	-	100010916	-	3562	612.55616
palmitoyl-oleoyl-glycerol (16:0/18:1) [2]*	LC/MS pos late	54942	-	-	-	100010917	-	3695	612.55616
palmitoleoyl-oleoyl-glycerol (16:1/18:1) [1]*	LC/MS pos late	52632	-	HMDB07131	9543694	100009053	-	3035	610.54051
palmitoleoyl-oleoyl-glycerol (16:1/18:1) [2]*	LC/MS pos late	52631	-	-	-	100009054	-	3154	610.54051
palmitoleoyl-palmitoleoyl-glycerol (16:1/16:1) [2]*	LC/MS pos late	57409	-	-	-	100015686	-	2708	582.50921
palmitoyl-palmitoyl-glycerol (16:0/16:0) [1]*	LC/MS pos late	54991	-	-	-	100010947	-	3568	586.54051
palmitoyl-palmitoyl-glycerol (16:0/16:0) [2]*	LC/MS pos late	54990	-	-	-	100010948	-	3734	586.54051
palmitoyl-linoleoyl-glycerol (16:0/18:2) [2]*	LC/MS pos late	52634	-	-	-	100009055	-	3197	610.54051
palmitoyl-linolenoyl-glycerol (16:0/18:3) [2]*	LC/MS pos late	54965	-	-	-	100010939	-	2834	608.52486
stearoyl-linoleoyl-glycerol (18:0/18:2) [2]*	LC/MS pos late	54947	-	-	-	100010921	-	3762	638.57181
stearoyl-linolenoyl-glycerol (18:0/18:3) [2]*	LC/MS pos late	57326	-	-	-	100015585	-	3317	636.55616
oleoyl-oleoyl-glycerol (18:1/18:1) [1]*	LC/MS pos late	54945	-	-	-	100010918	-	3571	638.57181
oleoyl-oleoyl-glycerol (18:1/18:1) [2]*	LC/MS pos late	54946	-	-	-	100010919	-	3695	638.57181
sphinganine	LC/MS pos late	17769	C00836	HMDB00269	3126	313	3102-56-5	1413	302.30536

3-ketosphinganine	LC/MS pos late	34381	C02934	HMDB01480	631	100001436	18944-28-0	1400	300.28971
N-palmitoyl-sphinganine (d18:0/16:0)	LC/MS pos late	52604	-	HMDB11760	5283572	100009028	5966-29-0	3090	540.53503
N-palmitoyl-sphingadienine (d18:2/16:0)*	LC/MS pos late	57416	-	-	-	100015609	-	2490	536.50372
N-behenoyl-sphingadienine (d18:2/22:0)*	LC/MS pos late	57372	-	-	-	100015624	-	3911	620.59763
myristoyl dihydrosphingomyelin (d18:0/14:0)*	LC/MS pos late	57365	-	-	-	100009038	-	2060	677.5592
palmitoyl dihydrosphingomyelin (d18:0/16:0)*	LC/MS pos late	52434	-	-	9939965	100008954	-	2290	705.5905
behenoyl dihydrosphingomyelin (d18:0/22:0)*	LC/MS pos late	57331	-	-	-	100009026	-	3150	789.68441
palmitoyl sphingomyelin (d18:1/16:0)	LC/MS pos late	37506	-	-	9939941	100002107	6254-89-3	2168	703.57485
stearoyl sphingomyelin (d18:1/18:0)	LC/MS pos late	19503	C00550	HMDB01348	6453725	1538	85187-10-6;85187-10-6	2400	731.60615
behenoyl sphingomyelin (d18:1/22:0)*	LC/MS pos late	48492	-	-	-	100006294	-	3083	787.66876
tricosanoyl sphingomyelin (d18:1/23:0)*	LC/MS pos late	52436	-	-	-	100008955	-	3200	801.68441
lignoceroyl sphingomyelin (d18:1/24:0)	LC/MS pos late	57330	-	-	-	100006298	60037-60-7	3437	815.70006
sphingomyelin (d18:1/14:0, d16:1/16:0)*	LC/MS pos late	42463	-	-	11433862	100004328	-	1998	675.54355
sphingomyelin (d18:2/14:0, d18:1/14:1)*	LC/MS pos late	47154	-	-	-	100005985	-	1860	673.5279
sphingomyelin (d18:1/15:0, d16:1/17:0)*	LC/MS pos late	52433	-	-	-	100006314	121999-58-4	2082	689.5592
sphingomyelin (d18:2/16:0, d18:1/16:1)*	LC/MS pos late	42459	-	-	-	100004329	-	2002	701.5592
sphingomyelin (d18:1/17:0, d17:1/18:0, d19:1/16:0)	LC/MS pos late	52615	-	-	-	100008920	121999-64-2	2312	717.5905
sphingomyelin (d18:1/18:1, d18:2/18:0)	LC/MS pos late	37529	-	-	6443882	100002106	108392-10-5	2167	729.5905
sphingomyelin (d18:1/20:0, d16:1/22:0)*	LC/MS pos late	48490	-	-	-	100006290	-	2685	759.6375
sphingomyelin (d18:1/20:1, d18:2/20:0)*	LC/MS pos late	48491	-	-	-	100006292	222403-67-0	2383	757.6218
sphingomyelin (d18:1/21:0, d17:1/22:0, d16:1/23:0)*	LC/MS pos late	52495	-	-	-	100009025	-	2793	773.65311
sphingomyelin (d18:1/22:1, d18:2/22:0, d16:1/24:1)*	LC/MS pos late	48493	-	-	-	100006295	-	2666	785.65311
sphingomyelin (d18:2/23:0, d18:1/23:1, d17:1/24:1)*	LC/MS pos late	52435	-	-	-	100008956	-	2845	799.66868
sphingomyelin (d18:1/24:1, d18:2/24:0)*	LC/MS pos late	47153	-	-	-	100005986	94359-13-4	3033	813.68441
sphingomyelin (d18:2/24:1, d18:1/24:2)*	LC/MS pos late	52437	-	-	-	100008957	-	2635	811.66868
sphingosine	LC/MS pos late	17747	C00319	HMDB00252	5353955	297	123-78-4	1393	300.28971
sphingosine 1-phosphate	LC/MS pos late	34445	C06124	HMDB00277	5283560	100000626	26993-30-6	1375	380.25604
N-myristoyl-sphingosine (d18:1/14:0)*	LC/MS pos late	57366	C13916	HMDB11773	5282310	100015608	-	2479	510.48807
N-palmitoyl-sphingosine (d18:1/16:0)	LC/MS pos late	44877	-	HMDB04949	5283564	1518	24696-26-2	2893	538.51937
N-margaroyl-sphingosine (d18:1/17:0)*	LC/MS pos late	57367	-	-	-	100015611	-	3054	552.53503
N-stearoyl-sphingosine (d18:1/18:0)*	LC/MS pos late	54979	-	HMDB04950	5283565	1547	104404-17-3	3392	566.55067
N-stearoyl-sphingadienine (d18:2/18:0)*	LC/MS pos late	57417	-	-	-	100015632	-	2846	564.53503
phytosphingosine	LC/MS pos late	1510	C12144	HMDB04610	122121	469	554-62-1	1367	318.30027
glycosyl-N-palmitoyl-sphingosine (d18:1/16:0)	LC/MS pos late	53013	-	-	-	100009272	-	2623	700.5722
glycosyl-N-stearoyl-sphingosine (d18:1/18:0)	LC/MS pos late	52234	-	-	-	100001882	-	3053	728.6035
glycosyl-N-behenoyl-sphingadienine (d18:2/22:0)*	LC/MS pos late	57421	-	-	-	100015625	-	3420	782.65045
glycosyl-N-nervonoyl-sphingosine (d18:1/24:1)*	LC/MS pos late	57369	-	-	-	100001878	-	3990	810.68175
glycosyl-N-(2-hydroxynervonoyl)-sphingosine (d18:1/24:1(2OH))*	LC/MS pos late	57444	-	-	-	100015752	-	3839	826.67665
glycosyl-N-stearoyl-sphinganine (d18:0/18:0)*	LC/MS pos late	57418	-	-	6321364	100015694	-	3134	730.61915

lactosyl-N-palmitoyl-sphingosine (d18:1/16:0)	LC/MS pos late	53010	-	-	-	100009030	4201-62-1	2527	862.62502
lactosyl-N-stearoyl-sphingosine (d18:1/18:0)*	LC/MS pos late	54980	-	HMDB11591	10260120	100010945	-	2906	890.65632
lactosyl-N-nervonoyl-sphingosine (d18:1/24:1)*	LC/MS pos late	57370	-	-	-	100015620	-	3778	972.73457
sphingomyelin (d18:2/23:1)*	LC/MS pos late	57482	-	-	-	100015791	-	2315	797.65308
sphingomyelin (d18:2/24:2)*	LC/MS pos late	57479	-	-	-	100015789	-	2215	809.65308
sphingomyelin (d18:1/25:0, d19:0/24:1, d20:1/23:0, d19:1/24:0)*	LC/MS pos late	57478	-	-	-	100015792	-	3220	829.71571
sphingomyelin (d18:1/22:2, d18:2/22:1, d16:1/24:2)*	LC/MS pos late	57477	-	-	-	100006296	-	2209	783.63746
sphingomyelin (d18:0/20:0, d16:0/22:0)*	LC/MS pos late	57476	-	-	-	100015786	-	2600	761.65311
sphingomyelin (d18:0/18:0, d19:0/17:0)*	LC/MS pos late	57473	-	-	-	100009027	-	2362	733.6218
sphingomyelin (d17:2/16:0, d18:2/15:0)*	LC/MS pos late	57483	-	-	-	100015793	-	1837	687.54355
sphingomyelin (d18:1/19:0, d19:1/18:0)*	LC/MS pos late	57475	-	-	-	100015787	-	2330	745.62178
N-palmitoyl-heptadecasphingosine (d17:1/16:0)*	LC/MS pos late	57430	-	-	-	100015731	-	2645	524.50374
N-stearoyl-sphinganine (d18:0/18:0)*	LC/MS pos late	1759	-	-	5283573	922	2304-80-5	3526	568.56632
glycosyl-N-tetracosadienoyl-sphingosine (d18:1/24:2)*	LC/MS pos late	57452	-	-	-	100015703	-	3380	808.66608
3-hydroxy-3-methylglutarate	LC/MS polar	531	C03761	HMDB00355	1662	112	503-49-1	2700	161.04555
cholesterol	LC/MS pos late	63	C00187	HMDB00067	11025495	266	57-88-5	2707	369.35159
7-dehydrocholesterol	LC/MS pos late	1099	C01164	HMDB00032	439423	264	434-16-2	2467	367.33594
campesterol	LC/MS pos late	33997	C01789	HMDB02869	173183	100001269	474-62-4	2873	383.36724
7-hydroxycholesterol (alpha or beta)	LC/MS pos late	47890	-	-	-	100005999	-	1930	367.33594
cholate	LC/MS neg	22842	C00695	HMDB00619	221493	136	81-25-4	5165	407.28029
glycocholate	LC/MS neg	18476	C01921	HMDB00138	10140	342	475-31-0;863-57-0	5163	464.30176
taurocholate	LC/MS neg	18497	C05122	HMDB00036	6675	1648	145-42-6	5150	514.28439
chenodeoxycholate	LC/MS neg	1563	C02528	HMDB00518	10133	1123	474-24-9;474-25-9	5264	391.28538
glycochenodeoxycholate	LC/MS neg	32346	C05466	HMDB00637	12544	1628	16564-43-5	5236.1	448.30684
taurochenodeoxycholate	LC/MS neg	18494	C05465	HMDB00951	387316	1629	6009-98-9	5250	498.28948
deoxycholate	LC/MS neg	1114	C04483	HMDB00626	222528	302	83-44-3	5294	391.28538
taurodeoxycholate	LC/MS neg	12261	C05463	HMDB00896	2733768	1668	207737-97-1	5257.4	498.28948
ceramide (d16:1/24:1, d18:1/22:1)*	LC/MS pos late	57437	-	-	-	100015727	-	3850	620.59763
ceramide (d18:1/20:0, d16:1/22:0, d20:1/18:0)*	LC/MS pos late	57440	-	-	-	100015755	-	3920	594.58199
ceramide (d18:2/24:1, d18:1/24:2)*	LC/MS pos late	57443	-	-	-	100015744	-	3858	646.6133
glycosyl ceramide (d16:1/24:1, d18:1/22:1)*	LC/MS pos late	57457	-	-	-	100015730	-	3373	782.65045
glycosyl ceramide (d18:1/20:0, d16:1/22:0)*	LC/MS pos late	57595	-	-	-	100015882	-	3414	756.6348
glycosyl ceramide (d18:1/23:1, d17:1/24:1)*	LC/MS pos late	57448	-	-	-	100015751	-	3667	796.66609
eicosanoylsphingosine (d20:1)*	LC/MS pos late	57597	-	-	-	100015870	-	1443	328.321
palmitoylcholine	LC/MS pos late	52944	-	-	151731	100009233	-	1482	342.33666
palmitoloelycholine	LC/MS pos late	53257	-	-	-	100009334	-	1385	340.32101
oleoylcholine	LC/MS pos late	53260	-	-	-	100009331	-	1449	368.35231
linoleoylcholine*	LC/MS pos late	57463	-	-	-	100015760	-	1388	366.33666
stearoylcholine*	LC/MS pos late	57464	-	-	-	100015759	-	1520	370.36797
inosine 5'-monophosphate (IMP)	LC/MS polar	2133	C00130	HMDB00175	8582	362	4691-65-0	4267.7	347.03982
inosine	LC/MS pos early	1123	C00294	HMDB00195	6021	361	58-63-9	1269	269.08805

hypoxanthine	LC/MS neg	3127	C00262	HMDB00157	790	171	68-94-0	1291.2	135.03123
xanthine	LC/MS neg	3147	C00385	HMDB00292	1188	1004	69-89-6	910	151.02615
xanthosine 5'-monophosphate (xmp)	LC/MS neg	12024	C00655	HMDB01554	73323	1251	25899-70-1	580	363.03474
xanthosine	LC/MS neg	15136	C01762	HMDB00299	64959	100000299	146-80-5	1075	283.0684
N1-methylinosine	LC/MS pos early	48351		HMDB02721	65095	100001409	20245-33-4	1430	283.1037
2'-deoxyinosine	LC/MS neg	15076	C05512	HMDB00071	65058	100000135	890-38-0	1688	251.07857
urate	LC/MS neg	1604	C00366	HMDB00289	1175	1134	69-93-2;120K5305	757.1	167.02106
allantoin	LC/MS polar	1107	C02350	HMDB00462	204	1002	97-59-6	1672	157.03671
adenosine 5'-diphosphate (ADP)	LC/MS neg	3108	C00008	HMDB01341	6022	208	20398-34-9	680	426.02214
adenosine 5'-monophosphate (AMP)	LC/MS pos early	32342	C00020	HMDB00045	6083	209	149022-20-8	1013	348.07037
adenosine 3'-monophosphate (3'-AMP)	LC/MS neg	35142	C01367	HMDB03540	41211	100001449	84-21-9	1245	346.0558
adenosine 2'-monophosphate (2'-AMP)	LC/MS neg	36815	C00946	HMDB11617	94136	100001694	130-49-4	1497.7	346.0558
adenosine 3',5'-cyclic monophosphate (cAMP)	LC/MS neg	2831	C00575	HMDB00058	6076	207	60-92-4	1952.2	328.04524
adenylosuccinate	LC/MS neg	18360	C03794	HMDB00536	195	1303	19046-78-7	560	230.52974
adenosine	LC/MS pos early	555	C00212	HMDB00050	60961	798	58-61-7	2169	268.10404
adenine	LC/MS pos early	554	C00147	HMDB00034	190	880	73-24-5	2221	136.06178
N1-methyladenosine	LC/MS pos early	15650	C02494	HMDB03331	27476	1242	15763-06-1	2120	282.11969
N6-methyladenosine	LC/MS pos early	37114		HMDB04044	102175	213	1867-73-8	2264	282.11969
N6,N6-dimethyladenosine	LC/MS pos early	42081			348206	100003867	2620-62-4	2415	296.13533
isopentenyl adenosine	LC/MS pos early	40466	C16427		266767	100003368	7724-76-7	3151	336.16663
N6-carbamoylthreonyladenosine	LC/MS neg	35157		HMDB41623	161466	100001415	24719-82-2	2164	411.12698
2'-deoxyadenosine 5'-diphosphate	LC/MS neg	15116	C00206	HMDB01508	188966	1228	72003-83-9	1050	410.02723
2'-deoxyadenosine 5'-monophosphate	LC/MS neg	46333	C00360	HMDB00905	12599	100000485	653-63-4	1315	330.06089
diadenosine triphosphate	LC/MS neg	47397	C06197	HMDB01155		100002712	56432-02-1	1213.7	755.07465
N6-succinyladenosine	LC/MS neg	48130		HMDB00912		100001664	4542-23-8	980	382.10043
guanosine 5'- diphosphate (GDP)	LC/MS neg	2848	C00035	HMDB01201	8977	346	43139-22-6	639	442.01706
guanosine 5'-monophosphate (5'-GMP)	LC/MS pos early	2849	C00144	HMDB01397	6804	347	12/9/50	800	364.06528
guanosine 3'-monophosphate (3'-GMP)	LC/MS polar	39786			3522	100002704	117-68-0	4210	362.05072
guanosine	LC/MS neg	1573	C00387	HMDB00133	6802	1099	118-00-3	1662	282.08439
guanine	LC/MS pos early	32352	C00242	HMDB00132	764	172	73-40-5	2032	152.05669
7-methylguanine	LC/MS pos early	35114	C02242	HMDB00897	11361	100001456	578-76-7	2175	166.07234
N2-methylguanosine	LC/MS neg	35133		HMDB05862	3035422	100001467	2140-77-4	1988	296.10004
N2,N2-dimethylguanosine	LC/MS pos early	35137		HMDB04824	92919	100001412	2140-67-2	2210	312.13025
2'-deoxyguanosine	LC/MS neg	1411	C00330	HMDB00085	187790	348	961-07-9	1751	266.08948
N-carbamoylaspartate	LC/MS neg	1594	C00438	HMDB00828	93072	1108	923-37-5	606.9	175.03604
dihydroorotate	LC/MS polar	601	C00337	HMDB03349	648	923	155-54-4	1910	157.02548
orotate	LC/MS polar	1505	C00295	HMDB00226	967	445	50887-69-9	1638.1	155.00983
orotidine 5'-phosphate	LC/MS polar	1506	C01103	HMDB00218	160617	446	68244-58-6	4500	367.01842
orotidine	LC/MS polar	35172	C01103	HMDB00788	92751	100001416	314-50-1	2250	287.05209
uridine 5'-triphosphate (UTP)	LC/MS neg	33448	C00075	HMDB00285	6133	871	19817-92-6	572	482.96126
uridine 5'-diphosphate (UDP)	LC/MS neg	5345	C00015	HMDB00295	6031	870	21931-53-3	599.5	402.99492

uridine 5'-monophosphate (UMP)	LC/MS polar	2856	C00105	HMDB00288	6030	869	3387-36-8	4150	323.02859
uridine 3'-monophosphate (3'-UMP)	LC/MS neg	39764	C01368	-	101543	106	35170-0-7;35170-03-7	750	323.02899
uridine	LC/MS neg	606	C00299	HMDB00296	6029	535	58-96-8	1457.6	243.06226
uracil	LC/MS polar	605	C00106	HMDB00300	1174	825	66-22-8	1089.7	111.02
pseudouridine	LC/MS neg	33442	C02067	HMDB00767	15047	821	1445-07-4	1100	243.06226
5-methyluridine (ribothymidine)	LC/MS neg	35136	-	HMDB00884	445408	100001446	1463-10-1	1778.1	257.07791
2'-deoxyuridine	LC/MS polar	1412	C00526	HMDB00012	13712	536	951-78-0	1100	273.07281
3-ureidopropionate	LC/MS pos early	3155	C02642	HMDB00026	111	1053	462-88-4	875	133.06077
beta-alanine	LC/MS pos early	55	C00099	HMDB00056	239	244	56-41-7;107-95-9	1905	90.05496
cytidine triphosphate	LC/MS neg	2844	C00063	HMDB00082	6176	281	36051-68-0	575	481.97724
cytidine diphosphate	LC/MS neg	2841	C00112	HMDB01546	6132	280	34393-59-4	580	402.01091
cytidine 5'-monophosphate (5'-CMP)	LC/MS pos early	2372	C00055	HMDB00095	6131	282	63-37-6	835	324.05914
cytidine	LC/MS pos early	514	C00475	HMDB00089	6175	827	65-46-3	2124	244.0928
3-methylcytidine	LC/MS pos early	35132	-	-	159649	100001466	21028-20-6	2176	258.10845
5-methylcytidine	LC/MS pos early	22119	-	HMDB00982	92918	100000763	2140-61-6	2227	258.10845
2'-deoxycytidine 5'-monophosphate	LC/MS pos early	533	C00239	HMDB01202	13945	298	1032-65-1	975	308.06422
2'-deoxycytidine	LC/MS pos early	15949	C00881	HMDB00014	13711	100000125	951-77-9	2193	228.09789
2'-O-methylcytidine	LC/MS pos early	57554	-	-	150971	100010895	2140-72-9	2411	258.10845
thymidine 5'-monophosphate	LC/MS neg	12023	C00364	HMDB01227	9700	1248	33430-62-5	883	321.04933
thymidine	LC/MS neg	2183	C00214	HMDB00273	5789	872	50-89-5	1963.7	241.08299
thymine	LC/MS neg	604	C00178	HMDB00262	1135	882	65-71-4	1654	125.03565
5,6-dihydrothymine	LC/MS pos early	1418	C00906	HMDB00079	93556	158	696-04-8	1280	129.06586
3-aminoisobutyrate	LC/MS pos early	1566	C05145	HMDB03911	64956	1114	10569-72-9;214139-20-5	2215	104.07061
methylphosphate	LC/MS polar	37070	-	HMDB61711	13130	100001805	7023-27-0	3760.1	110.98527
nicotinamide	LC/MS pos early	594	C00153	HMDB01406	936	432	98-92-0	1942	123.05529
nicotinamide ribonucleotide (NMN)	LC/MS pos early	22152	C00455	HMDB00229	14180	1312	1094-61-7	766	335.06388
nicotinamide riboside	LC/MS pos early	33013	C03150	HMDB00855	439924	100001310	1341-23-7	2040	255.09755
nicotinamide adenine dinucleotide (NAD+)	LC/MS neg	5278	C00003	HMDB00902	5893	1310	53-84-9	1434	662.10184
nicotinamide adenine dinucleotide reduced (NADH)	LC/MS neg	31475	C00004	HMDB01487	439153	428	58-68-4;606-68-8	1540	664.1175
nicotinamide adenine dinucleotide phosphate reduced (NADPH)	LC/MS neg	33450	C00005	HMDB00221	5884	430	2646-71-1	900	744.08383
1-methylnicotinamide	LC/MS pos early	27665	C02918	HMDB00699	10129985	55	1005-24-9	1940	137.07094
trigonelline (N'-methylnicotinate)	LC/MS pos early	32401	C01004	HMDB00875	5570	100001092	535-83-1	1388	138.05496
adenosine 5'-diphosphoribose (ADP-ribose)	LC/MS neg	558	C00301	HMDB01178	192	215	68414-18-6	920	558.06439
riboflavin (Vitamin B2)	LC/MS pos early	1827	C00255	HMDB00244	493570	500	83-88-5	2292	377.14557
flavin adenine dinucleotide (FAD)	LC/MS neg	2134	C00016	HMDB01248	643975	327	146-14-5;84366-81-4	2425	784.14985
flavin mononucleotide (FMN)	LC/MS neg	15797	C00061	HMDB01520	710	100000251	130-40-5	2390	455.09733
pantothenate	LC/MS pos early	1508	C00864	HMDB00210	6613	1024	137-08-6	1667	220.11795
phosphopantetheine	LC/MS pos early	15504	C01134	HMDB01416	987	100000328	NA	1372	359.10364
3'-dephosphocoenzyme A	LC/MS neg	18289	C00882	HMDB01373	444485	100000138	3633-59-8	2013	686.1416
3'-dephospho-acetyl-coenzyme A	LC/MS neg	54675	-	-	193680	100010805	-	2313	728.15216

coenzyme A	LC/MS neg	46322	C00010	HMDB01423	317	270	85-61-0;18439-24-2	1448	382.55033
pantetheine	LC/MS neg	57555	C00831	_	439322	100001495	496-65-1	2765	277.12275
threonate	LC/MS polar	27738	C01620	HMDB00943	151152	100001022	70753-61-6	2384	135.02989
gulonate*	LC/MS polar	46957	_	HMDB03290	9794176	100001586	20246-53-1	2750	195.05102
alpha-tocopherol	LC/MS pos late	1561	C02477	HMDB01893	14985	1105	59-02-9;10191-41-0	2522	430.37818
biotin	LC/MS pos early	568	C00120	HMDB00030	171548	251	58-85-5	2253	245.09545
folate	LC/MS neg	1826	C00504	HMDB00121	6037	328	59-30-3	1467	440.1324
5-methyltetrahydrofolate (5MeTHF)	LC/MS neg	18330	C00440	HMDB01396	146	1244	68703-91-3;68792-52-9	1820	458.17935
dihydrobiopterin	LC/MS pos early	35129	C00268	HMDB00038	1879	100001506	6779-87-9	1930	240.10912
pterin	LC/MS pos early	43023	C00715	HMDB00802	73000	100004216	22363-60-4	1805	164.05669
bilirubin (Z,Z)	LC/MS pos late	43807	C00486	HMDB00054	5280352	1090	635-65-4	1840	585.27076
thiamin (Vitamin B1)	LC/MS pos early	5341	C00378	HMDB00235	1130	873	59-43-8	3050	265.11176
thiamin monophosphate	LC/MS pos early	15798	C01081	HMDB02666	3382778	523	532-40-1	2378	345.07809
thiamin diphosphate	LC/MS neg	35670	C00068	HMDB01372	1132	522	154-87-0	1195.1	423.02986
retinol (Vitamin A)	LC/MS pos late	1806	C00473	HMDB00305	445354	498	68-26-8	1636	269.22639
pyridoxine (Vitamin B6)	LC/MS pos early	608	C00314	HMDB02075	1054	936	58-56-0	2442	170.08117
pyridoxamine	LC/MS pos early	2150	C00534	HMDB01431	1052	568	58052-48-5	2975	169.09716
pyridoxamine phosphate	LC/MS pos early	3138	C00647	HMDB01555	1053	493	529-96-4	1932	249.06349
pyridoxal phosphate	LC/MS pos early	5331	C00018	HMDB01491	1051	492	41468-25-1	1367	248.03186
pyridoxal	LC/MS pos early	1651	C00250	HMDB01545	1050	491	65-22-5	2343	168.06552
pyridoxate	LC/MS polar	31555	C00847	HMDB00017	6723	100001121	82-82-6	846	182.04588
hippurate	LC/MS neg	15753	C01586	HMDB00714	464	100000014	495-69-2	2106.9	178.05096
3-hydroxyhippurate	LC/MS neg	39600	_	HMDB06116	450268	100002122	1637-75-8	1687	194.04588
benzoate	LC/MS neg	15778	C00180	HMDB01870	243	100000008	65-85-0	1750	121.0295
catechol sulfate	LC/MS neg	35320	C00090	HMDB59724	3083879	100001605	4918-96-1	1906	188.98631
O-methylcatechol sulfate	LC/MS neg	46111	_	_	22473	100004208	_	2344	203.00196
4-methylcatechol sulfate	LC/MS neg	46146	_	_	_	100004111	_	2665	203.00196
4-ethylphenylsulfate	LC/MS neg	36099	C13637	_	_	100001756	123-07-9	3580	201.0227
gluconate	LC/MS polar	587	C00257	HMDB00625	10690	338	527-07-1	2922	195.05102
beta-guanidinopropanoate	LC/MS pos early	35101	C03065	HMDB13222	67701	100001411	353-09-3	2145	132.07676
ergothioneine	LC/MS pos early	37459	C05570	HMDB03045	3032311	100002154	58511-63-0	850	230.09578
erythritol	LC/MS polar	20699	C00503	HMDB02994	222285	100000846	149-32-6	1491	167.05611
homostachydrine*	LC/MS pos early	33009	C08283	HMDB33433	441447	100001550	1195-94-4	1750	158.11756
N-glycolylneuraminate	LC/MS pos early	37123	C03410	HMDB00833	123802	100001491	1113-83-3	636	326.10818
quinate	LC/MS polar	18335	C00296	HMDB03072	6508	100000442	77-95-2	2432.9	191.05611
stachydrine	LC/MS pos early	34384	C10172	HMDB04827	115244	100001296	4136-37-2	1440	144.10191
methyl glucopyranoside (alpha + beta)	LC/MS pos early	46144	_	_	_	100005864	_	832	212.11268
penicillin G	LC/MS pos early	37468	C05551	HMDB15186	5904	100002155	69-57-8	2870	335.10601
sulfate*	LC/MS neg	46960	C00059	HMDB01448	1118	100002528	14808-79-8	616	96.9601
O-sulfo-L-tyrosine	LC/MS neg	45413	_	_	514186	100005384	_	990	260.02343

2-aminophenol sulfate	LC/MS neg	43266	-	HMDB61116	181670	100004322	-	1677	188.0023
glycerol 2-phosphate	LC/MS polar	27728	C02979	HMDB02520	2526	100001029	819-83-0	3681.7	171.00639
HEPES	LC/MS neg	21248	-	-	23831	100000843	7365-45-9	1022	237.09145
lanthionine	LC/MS pos early	42002	-	-	6994972;98504	100003892	8/2/83	1730	209.05906
phenol red	LC/MS neg	36817	C12600	-	4766	100001724	143-74-8	3665.9	353.04891
benzoylcarnitine*	LC/MS pos early	43265	-	-	-	100004555	105450-08-6	3041	266.13869
4-acetamidobenzoate	LC/MS neg	57585	D03836	-	19266	100009413	556-08-1	1403	178.05096
